# Insights into recent findings and therapeutic potential of nonhistone lactylation in cancer

**DOI:** 10.3389/fmolb.2025.1661697

**Published:** 2025-09-09

**Authors:** Jing Jia, Na Liu, Xiaoren Zhu, Minbin Chen

**Affiliations:** ^1^ Department of Radiotherapy and Oncology, Affiliated Kunshan Hospital of Jiangsu University, Kunshan, China; ^2^ Department of Development and Regeneration, Stem Cell Institute, Katholieke Universiteit (KU) Leuven, Leuven, Belgium

**Keywords:** nonhistone, lactylation, post-translational modification, anti-tumor therapy, cancer metabolism

## Abstract

Lactylation, a recently identified post-translational modification, has become a crucial regulatory mechanism beyond its conventional metabolic role. Unlike histone lactylation, which regulates gene expression, nonhistone lactylation directly acts on effector proteins involved in processes such as signal transduction, metabolic reprogramming, and DNA damage repair. This article systematically reviews how nonhistone lactylation regulates biological processes related to cancer via mechanisms such as modulating protein interactions, stability, subcellular localization, and enzymatic activity. In addition, it comprehensively examines the potential applications and challenges in targeting nonhistone lactylation modification in antitumor treatment.

## 1 Introduction

In the 1920s, Otto Warburg discovered “aerobic glycolysis” (Urbano, 2021), a process in which cancer cells primarily utilize glycolysis despite oxygen availability (Vander Heiden et al., 2009). This phenomenon is known as the “Warburg effect” (Pouysségur et al., 2022) and is a hallmark of cancer. This metabolic shift considerably elevates lactate levels in the tumor microenvironment (Hanahan and Weinberg, 2011).

Once considered merely a metabolic waste product (Ganapathy-Kanniappan and Geschwind, 2013; Doherty and Cleveland, 2013), lactate’s biological significance was reconceptualized via the lactate shuttle theory (Brooks, 2018). Subsequent research revealed that lactate participates in energy metabolism and signal transduction and plays complex and crucial regulatory roles in tumor development and progression (Ye et al., 2022).

In 2019, Zhang et al. identified histone lactylation, a novel post-translational modification (PTM) in which lactyl groups attach to lysine residues (Zhang et al., 2019). This discovery transformed lactate’s role from a simple metabolic byproduct or signaling molecules to a significant modifier regulating gene expression and cellular behavior.

The initial studies focused on histone lactylation (Zhang et al., 2019; Yu et al., 2021; Zhang et al., 2024; Pan et al., 2022; Fan H. et al., 2023; Li et al., 2020; Sun et al., 2022); however, nonhistone lactylation far exceeds histone lactylation within cells in terms of variety and abundance (Sun et al., 2022). Hence, nonhistone lactylation may exert more profound effects on cellular function regulation than histone lactation. The targets of nonhistone lactylation include metabolic enzymes (Xiong et al., 2022), transcription factors (Wang X. et al., 2023), and DNA repair proteins (Chen Y. et al., 2024). By interacting with cancer-related genes and pathways, these lactylated proteins participate in crucial biological processes such as cancer cell metabolic regulation (Lu et al., 2024; Chen et al., 2025a; He et al., 2024), tumor immune microenvironment modulation (Ye et al., 2022; Dai et al., 2024; Gu et al., 2022; Yang H. et al., 2023), DNA repair (Chen Y. et al., 2024; Chen H. et al., 2024; Zong et al., 2024), and cellular autophagy (Sun W. et al., 2023; Jia et al., 2023; Meng et al., 2024; Huang Y. et al., 2024), influencing tumor development and treatment response (Xiong et al., 2022; Wang X. et al., 2023; Chen Y. et al., 2024; Lu et al., 2024; Gu et al., 2022; Yang H. et al., 2023; Chen H. et al., 2024).

This review focuses specifically on nonhistone lactylation—defined as lactylation modifications occurring on proteins other than histones (H1, H2A, H2B, H3, and H4)—and summarizes its molecular mechanisms in cancer development, emphasizing its regulatory roles in tumor metabolism, immune evasion, and therapeutic resistance. Furthermore, potential treatment strategies targeting nonhistone lactylation are explored, providing a strong theoretical foundation for developing novel anticancer drugs.

### 1.1 Lactylation modification mechanisms

**FIGURE 1:**
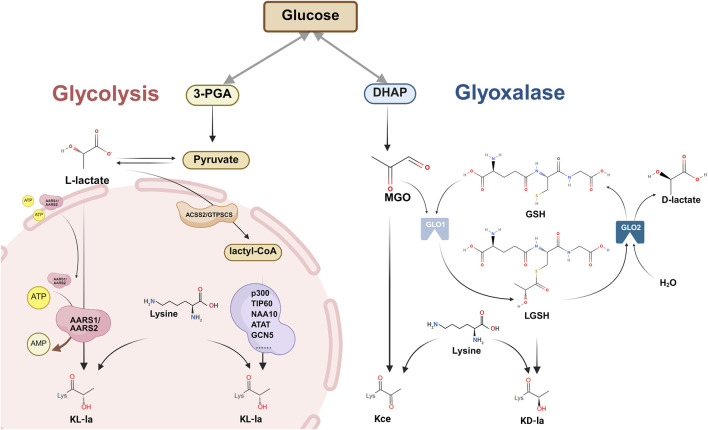
Mechanisms of lactylation modification: enzyme-dependent and non-enzymatic pathways. Overview of the two major pathways of protein lysine lactylation (Kla), involving the covalent attachment of lactyl groups to the ε-amino group of lysine residues. Three stereoisomeric Kla forms have been identified: L-lactyl-lysine (KL-la), D-lactyl-lysine (KD-la), and N-ε-(carboxyethyl)-lysine (Kce). Enzyme-dependent pathway: Pyruvate is converted to lactyl-CoA by ACSS2/GTPSCS enzymes. Multiple lysine acetyltransferases that function dually as lactyl transferases, transfer lactyl groups from lactyl-CoA to lysine residues to form L-lactyl-lysine (KL-la). The delactylase enzymes AARS1/AARS2 translocated to nucleus, reverse this modification via an ATP/AMP-dependent mechanism. Enzyme-independent pathway: DHAP generates MGO. MGO reacts with GSH under the action of GLO1 to form LGSH. LGSH directly transfers lactyl groups to lysine residues without enzymatic catalysis, forming KD-la. MGO can also directly modify lysine to generate Kce. GLO2 hydrolyzes LGSH to D-lactate in the presence of H_2_O. Abbreviations 3-PGA: 3-phosphoglyceric acid; ATP: adenosine triphosphate; AMP: adenosine monophosphate; ARRS1/ARRS2: alanine-tRNA synthetase 1/2; ACSS2: acyl-CoA synthetase short-chain family member 2; GTPSCS: GTP-specific succinate-CoA synthetase; DHAP: dihydroxyacetone phosphate; MGO: methylglyoxal; GSH: glutathione; LGSH: S-D-lactyl glutathione.

Lysine lactylation (Kla), a newly identified PTM (Zhang et al., 2019), involves the formation of a stable amide bond between lactic acid’s carboxyl group and the ε-amino group of lysine residues (Xu K. et al., 2024). Through analytical chemistry and mass spectrometry, researchers have distinguished the following three stereochemically distinct isomers: L-lactyl-lysine (KL-la), D-lactyl-lysine (KD-la), and N-ε(carboxyethyl)-lysine (Kce) (Zhang et al., 2025). Current research revealed two distinct pathways of lactylation: non-enzymatic and enzyme-dependent mechanisms (Figure 1). A detailed description of each mechanism is provided below.

#### 1.1.1 Non-enzymatic dependent mechanism

Gaffny’s 2020 discovery revealed that non-enzymatic Kla can occur through lactoylglutathione (LGSH)—a metabolite produced via the glutathione pathway (Gaffney et al., 2020). LGSH is generated through the glutathione pathway (Wu et al., 2004), primarily involving two key enzymes: glyoxalase (GLO) 1/2. This process begins when methylglyoxal (MGO), a glycolytic byproduct, reacts with glutathione (GSH) in the presence of GLO1 to form LGSH, which GLO2 later hydrolyzes into D-lactate and GSH (Wu et al., 2004). Past studies have demonstrated that LGSH transfers its acyl group to protein lysine residues without any apparent enzyme catalysis, as supported by observations that GLO2 knockout increased both the LGSH concentration and lactylation levels (Gaffney et al., 2020). Galligan et al. further demonstrated that LGSH could spontaneously convert to D-lactyl-CoA through S-to-S acyl transfer, thereby providing lactyl groups for KD-la formation without any enzymatic involvement (Zhao et al., 2025) (Figure 1). Although these studies did not completely exclude potential writer enzymes in KD-la formation, they established an LGSH concentration as a key driver of lactylation modification.

MGO’s high reactivity allows it to interact with diverse protein residues including arginine, cysteine, and lysine. In histones, lysine residues can form Kce (Figure 1), although at significantly lower levels compared to MGO-derived arginine modifications (Galligan et al., 2018). Similar to KD-la, this modification occurs non-enzymatically (Gaffney et al., 2020; Galligan et al., 2018).

#### 1.1.2 Enzyme-dependent mechanism

D-lactate constitutes only 1%–5% of total lactate in mammals, while L-lactate predominates in humans and eukaryotes, having been sourced from diet, gut bacteria, or the MGO pathway (Levitt and Levitt, 2020; Bianchetti et al., 2018). KL-la is confirmed as the earliest and the most dominant lactylation isomer (Zhang et al., 2019; Zhang et al., 2025). It operates through an enzyme-dependent mechanism (Zhang et al., 2019) involving three classes of key proteins: writers, readers, and erasers. Writers catalyze lactyl group to lysine residues. Readers specifically recognize and bind to lactyl groups. Erasers (delactylases) hydrolyze lactyl groups, that is, they remove lactylation modifications and restore the target molecules to their original states (Zhang et al., 2025).

### 1.2 Writers and Lactyl-CoA synthetases

Lactylation and acetylation share significant similarities: both derive primarily from pyruvate, possess similar molecular structures, and target lysine residues (Liu et al., 2023; Sabari et al., 2017; Stacpoole and Dirain, 2024). Owing to these structural and functional parallels, early lactylation research recommended lactyl-CoA as the substrate for this modification (Zhang et al., 2019). Initial investigations identified several acetyltransferases that function dually as lactyl transferases, including p300, CREB-binding protein (CBP), KAT2A, KAT5/TIP60, HBO1 (KAT7), KAT8, NAA10, ATAT1, and GCN5 (Wang X. et al., 2023; Chen Y. et al., 2024; Chen H. et al., 2024; Huang Y. et al., 2024; Fan M. et al., 2023; Hu et al., 2024; Huang H. et al., 2024; Liu et al., 2025; Moreno-Yruela et al., 2022; Wang et al., 2024; Yang et al., 2022; Yang et al., 2024; Zhang N. et al., 2023; Chen J. et al., 2024; Zhu et al., 2025; Chen et al., 2025b; Niu et al., 2024; Xie et al., 2024; Yuan et al., 2025; Niu et al., 2025; Yan et al., 2024; Cai et al., 2011; Wang N. et al., 2022; Tong et al., 2024). These writers demonstrated selectivity during lactylation catalysis, suggesting that different proteins’ lactylation may involve distinct catalytic enzymes, with lactyl-CoA majorly serving as the lactyl group donor (Gao et al., 2024). However, the field of lactyl-CoA synthetase research remained relatively underexplored.

A breakthrough occurred in November 2024 when (Zhu et al., 2025) identified acyl-CoA synthetase short-chain family member 2 (ACSS2) as the first mammalian lactyl-CoA synthetase (Zhu et al., 2025). When phosphorylated at S267, ACSS2 translocates to the nucleus where it converts lactate dehydrogenase (LDH) A -produced lactate into lactyl-CoA. ACSS2 then complexes with KAT2A, which then uses this lactyl-CoA as a substrate to complete histone lactylation (Zhu et al., 2025). Subsequently, Liu et al. discovered that GTP-specific succinate-CoA synthetase (GTPSCS) can relocate from the mitochondria to the nucleus, where it interacts with p300. Within this complex, GTPSCS generates lactyl-CoA *in situ* from lactate, while p300 mediates KL-la (Figure 1), thereby ultimately promoting glioblastoma progression through the GTPSCS/p300/H3K18la/GDF15 signaling axis (Liu et al., 2025). Despite these discoveries, lactyl-CoA concentration in cancer cells remains approximately 1000-fold lower than acetyl-CoA (Ju et al., 2024), which potentially limits the activity of lactyl transferases that utilize lactyl-CoA as a substrate. This significant disparity highlights the importance of further research to identify additional lactyl transferases and elucidate their catalytic mechanisms.

In 2023, Sun’s research on gastric cancer revealed that elevated copper levels enhanced interaction between alanine-tRNA synthetase (AARS) 1/2 and methyltransferase-like protein (METTL)16. Silencing AARS1/2 effectively suppressed copper-induced lactylation at K229 of METTL16. *In vitro* lactylation assay demonstrated that lactylation at METTL16 K229 was mediated by AARS1/2, same effect absented in METTL16 K229R mutant, suggesting AARS1/2 might mediate METTL16 K229 lactylation (Sun L. et al., 2023). By January 2024, Mao et al. observed structural similarities between lactate and alanine, which led them to hypothesize that alanyl-tRNA synthetases may recognize lactate. Their experiments confirmed AARS2’s ability to catalyze lactylation of mitochondrial proteins pyruvate dehydrogenase E1 alpha 1 (PDHA1) at K336 and carnitine palmitoyltransferase 2 (CPT2) at K457/458 (Mao et al., 2024). In March 2024, Ju et al. identified AARS1 as a *bona fide* lactyl transferase with multiple functions. Under ATP-dependent conditions, AARS1 senses lactate levels, translocate to the nucleus and directly uses lactate as a lactyl group donor to catalyze Yes-associated protein (YAP) and transcriptional enhanced associate domain (TEAD) lactylation in the Hippo pathway, which activates downstream gene transcription (Ju et al., 2024). Simultaneously, Zong demonstrated that AARS1 directly binds lactate and promotes p53 lactylation (K120/K139). The mechanism involves ATP activating lactate within AARS1 to form lactate-adenosine monophosphate, which then covalently attaches to lysine residues while releasing AMP. Among the AARS family, only AARS1-depleted cell lysates failed to lactylate p53, thereby establishing AARS1 as the primary p53 lactyl transferase (Zong et al., 2024). Further lactylation proteomics analysis confirmed AARS1/AARS2 as lactate-sensing proteins with lactyl transferase activity and identified that AARS2 catalyzed cyclic GMP-AMP synthase (cGAS) lactylation, which decreased cGAMP and interferon-β levels and suppressed innate immune responses (Li H. et al., 2024). The balance between AARS1/AARS2’s lactylation and alanine transfer functions involves competitive inhibition: alanine inhibits AARS’s lactylation activity, with β-alanine pretreatment dramatically reducing the lactylation levels in gastric cancer cells. Conversely, increased lactate competitively inhibits alanine function, while AARS1-mediated YAP lactylation enhances the AARS expression through positive feedback, thereby improving both lactate modification and alanine transport functions (Zong et al., 2024; Ju et al., 2024).

### 1.3 Readers

The first evidence of lactylation readers was reported by a study on induced pluripotent stem cell (iPSC) reprogramming, which revealed Brahma-related gene 1’s binding to H3K18 lactylation, thereby confirming its role as a lactylation reader (Hu et al., 2024). Subsequent research in cervical cancer, using multivalent photoaffinity probes with quantitative proteomics, identified Double PHD Fingers 2 as a specific reader of H3K14 lactylation (Zhai et al., 2024). In addition, AlphaScreen technology screening of 28 human bromodomain proteins discovered that tripartite motif (TRIM) 33 could specifically recognize multiple lactylation sites (Nuñez et al., 2024).

### 1.4 Eraser

Previous studies have identified several erasers for lactylation modification, primarily histone deacetylase (HDAC) one to three and sirtuin (SIRT) 1–3 (Chen Y. et al., 2024; Moreno-Yruela et al., 2022; Jin et al., 2023). Zessin et al. discovered that HDAC6 and HDAC8 also exhibit potential delactylase activity, although their enzymatic activity is lower than that of HDAC3 (Zessin et al., 2022). All these enzymes have been primarily known for their deacetylase activities, although now they are recognized to possess dual functionality in removing both acetyl and lactyl groups from proteins. This functional duality raises questions regarding their enzymatic properties and biological roles in lactylation dynamics.

Lactylation, as a reversible PTM, provides cells with a dynamic and precise mechanism for regulating protein function. However, the full landscape of lactylation-modifying enzymes—writers, readers, erasers, and lactyl-CoA synthetases—remains unclear. Due to the unclear specific conditions through which lactylation-modification enzymes exert lactylation-related functions, future research may focus on comprehensively elucidating the functional specificity, substrate specificity, regulatory mechanisms of lactylation-modification enzymes, and their roles in tumorigenesis.

## 2 Lactylation predominantly occurs in nonhistones

Recent advances in lactylomics have revealed that lactylation modifications predominantly occur in nonhistone proteins and are upregulated in tumor tissues (Table 1). Several studies have provided compelling evidence for this distribution pattern. Chen performed lactylome analysis of six pairs of non-small cell lung cancer tissues and matched normal adjacent tissues. The study identified 2,193 Kla sites on 806 proteins, with 97.8% (2,144 sites) occurring on nonhistone proteins (Chen J. et al., 2024). Yang et al. conducted a comprehensive lactylome analysis of hepatitis B virus (HBV)-associated hepatocellular carcinoma cohorts. This study identified 9,275 lactylation sites, of which an overwhelming 99.8% (9,256 sites) were located on nonhistone proteins (Yang Z. et al., 2023). Duan’s integrated lactylome analysis of 40 pairs of gastrointestinal tumor tissues and normal adjacent tissues revealed 11,698 lactylation sites on 3,156 proteins, of which 98.9% (11,571 sites) were located on nonhistone proteins (Duan et al., 2024).

**TABLE 1:** Summary of Known lactylation modified Nonhistone.

Protein	Lactylation Site(s)	Primary function	Effect of lactylation	References
Metabolic Enzymes				
METTL3	K281/K345	m6A RNA methylation	Enhanced RNA binding affinity	Xiong et al. (2022)
ALDOA	K230/K322; K147	Glycolysis	Weakened binding affinity with DEAD-box helicase 17; reduced enzymatic activity	Feng et al. (2024), Shao et al. (2025), Wan et al. (2022)
PFKP	K392	Glycolysis	Suppressed PTEN expression	Mi (2024)
PCK2	K100	Gluconeogenesis	Enhanced enzymatic activity	Yuan et al. (2025)
PDHA1	K336	Pyruvate metabolism	Inactivated enzyme	Mao et al. (2024)
CPT2	K457/K458	Fatty acid oxidation	Inactivated enzyme	Mao et al. (2024)
NMNAT1	K128	NAD + synthesis	Maintained enzymatic activity	Huang et al. (2024b)
NSUN2	K508	RNA methylation	Enhanced catalytic activity	Niu et al. (2025)
METTL16	K229	RNA methylation	Increased enzymatic activity	Sun et al. (2023b)
Transcription Factors				
p53	K120/K139	Tumor suppressor	Disrupted DNA binding	Zong et al. (2024)
YY1	K138; K183	Transcription regulation	Enhanced promoter binding; increased FBXO33 transcription	Wang et al. (2023a), Wu et al. (2025)
YAP	Multiple sites	Hippo pathway	Enhanced transcriptional activity	Ju et al. (2024)
TEAD1	Multiple sites	Hippo pathway	Enhanced transcriptional activity	Ju et al. (2024)
TFEB	K91	Autophagy regulation	Inhibited WWP2 interaction	Huang et al. (2024a)
HIF-1α	Multiple sites	Hypoxia response	Enhanced stability	Luo et al. (2022)
DNA Repair Proteins				
MRE11	K673/K678	DNA repair	Enhanced DNA binding and repair	Chen et al. (2024a)
NBS1	K388	DNA repair	Promoted MRE11 complex formation	Chen et al. (2024b)
XRCC1	K247	DNA repair	Enhanced nuclear translocation	Li et al. (2024b)
RNA-Binding Proteins				
IGF2BP3	K76	RNA stability	Enhanced m6A-RNA binding	Lu et al. (2024)
PTBP1	K436	RNA splicing	Disrupted TRIM21 interaction	Zhou et al. (2025)
Nucleolin	K477	RNA processing	Enhanced RNA binding	Yang et al. (2024)
Signaling Proteins				
β-catenin	Multiple sites	Wnt signaling	Enhanced stability	Miao et al. (2023)
MOESIN	K72	Cell adhesion	Enhanced TGF-β receptor interaction	Gu et al. (2022)
HMGB1	Near NLS	DNA binding/signaling	Promoted cytoplasmic translocation	Yang et al. (2022)
PD-L1	K810-813	Immune checkpoint	Inhibited lysosomal degradation	Tong et al. (2024)
Other Proteins				
APOC2	K70	Lipid metabolism	Enhanced protein stability	Chen et al. (2024c)
CEACAM6	Multiple sites	Cell adhesion	Enhanced stability	Chu et al. (2023)
NUSAP1	Multiple sites	Cell cycle	Enhanced stability	Chen et al. (2023)
DCBLD1	K172	Signal transduction	Inhibited ubiquitination	Meng et al. (2024)
ABCF1	K430	ABC transporter	Enhanced nuclear translocation	Hong et al. (2025)
eEF1A2	K408	Protein synthesis	Enhanced GTPase activity	Xie et al. (2024)
TUFM	K286	Mitochondrial translation	Impaired mitochondrial localization	Weng et al. (2025)
Cyclin E2	Multiple sites	Cell cycle	Enhanced stability	Jin et al. (2023)
cGAS	Multiple sites	Innate immunity	Decreased enzymatic activity	Li et al. (2024a)
Adenylate kinase 2	K28	Nucleotide metabolism	Decreased kinase activity	Yang et al. (2023b)

Cell-level investigations have yielded similar findings. For instance, James identified 350 lactylated proteins in their preliminary studies on nonenzymatic reactions of lactyl-glutathione (Gaffney et al., 2020). In another research, Lan et al. identified 1,569 lactylation sites on 799 proteins in pancreatic cancer cell lines (Huang H. et al., 2024). Lactylome analysis of the human gastric cancer cell line HGC27 identified 2,789 Kla sites on 1,182 proteins (Ju et al., 2024). Considering the limited variety of histones (primarily H2A, H2B, H3, and H4), these findings establish that lactylation is a common PTM that predominantly occurs in nonhistone proteins. Although early lactylation research focused on histones and epigenetic regulation, emerging studies demonstrate that most lactylation modifications occur in nonhistone proteins.

## 3 Impact of lactylation modifications on the functions of nonhistones

Unlike histone lactylation that primarily regulates gene transcription through chromatin modifications, nonhistone lactylation directly modulates protein function through conformational changes, stability alterations, and subcellular localization.

### 3.1 Protein molecular interactions

Lactylation modifies to specify protein-RNA interactions by altering protein conformation and surface charge. In N6-methyladenosine (m6A) RNA modification, K281la and K345la within the zinc finger domain of METTL3 considerably augment its binding affinity for m6A-modified RNA (Xiong et al., 2022). Similarly, insulin-like growth factor 2 mRNA-binding protein 3 (IGF2BP3) K76la strengthens its binding to m6A-modified phosphoenolpyruvate carboxykinase 2 (PCK2) and nuclear factor erythroid 2-related factor 2 mRNAs, triggering serine metabolism reprogramming (Lu et al., 2024). Furthermore, in intrahepatic cholangiocarcinoma, nucleolin K477la promotes target RNA binding via conformational changes, subsequently regulating downstream gene expression (Yang et al., 2024).

In addition, lactylation influences protein–DNA interactions. ATP-binding cassette transporter F1 (ABCF1) K430la mediates its nuclear translocation and enhances its binding to the lysine demethylase 3A (KDM3A) promoter, upregulating its expression (Hong et al., 2025). Meiotic recombination 11 (MRE11) K678la improves DNA binding and stimulates DNA end resection and homologous recombination repair efficiency without affecting the formation of the MRE11-RAD50-NBS complex (Chen Y. et al., 2024). Yin Yang-1 (YY1) K138la increases the binding to fibroblast growth factor 2 promoter, upregulating the transcription of fibroblast growth factor 2 (Wang X. et al., 2023). Similarly, YY1 K138la accentuates its binding to the FBXO33 promoter, substantially upregulating FBXO33 mRNA and protein expression (Wu et al., 2025).

**FIGURE 2:**
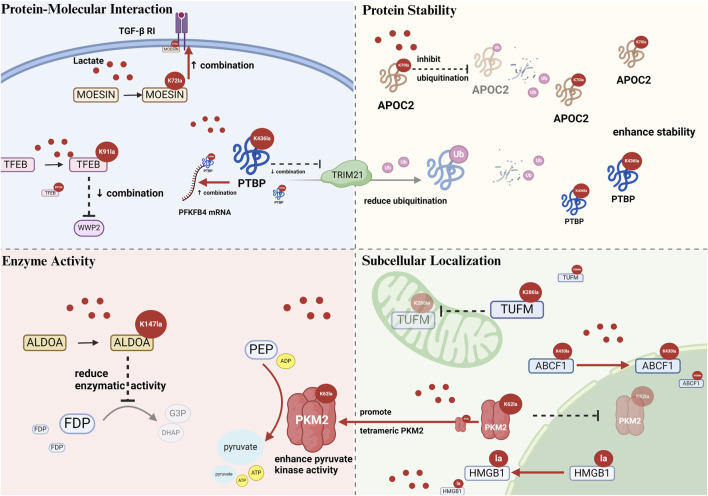
Schematic illustration of alterations caused by nonhistone lactylation on protein–protein interaction, stability, enzymatic activity, and subcellular localization. *Protein–molecular interactions*: Kla modulates binding affinity with proteins or nucleic acids, e.g., MOESIN, TFEB, and PTBP1. *Protein stability*: Kla often inhibits ubiquitin-mediated proteins degradation, e.g., APOC2 K70la, PTBP1 K436la). *Enzyme activity*: Kla can enhance (PKM2 K62la) or inhibit (ALDOA K147la) enzymatic function. *Subcellular localization*: Kla alters intracellular trafficking, e.g., TUFM K286la inhibits mitochondrial entry, ABCF1 K430la enhances nuclear translocation. Abbreviations TGF-β RI: transforming growth factor-β type I receptor; MOESIN: membrane-organizing extension spike protein; TFEB: transcription factor EB; WWP2: WW domain containing E3 ubiquitin protein ligase 2; PTBP: polypyrimidine tract-binding protein 1; PFKFB4: 6-phosphofructo-2-kinase/fructose-2,6-biphosphatase 4; TRIM21: tripartite motif containing 21; APOC2: apolipoprotein C2; ALDOA: aldolase A; FDP: 1,6-fructose diphosphate; G3P: glyceraldehyde-3-phosphate; DHAP: dihydroxyacetone phosphate; PEPE: phosphoenolpyruvate; ADP: adenosine diphosphate; ATP: adenosine triphosphate; PKM2: pyruvate kinase M2; TUFM: mitochondrial elongation factor Tu; ABCF1: ATP-binding cassette transporter F1.

In addition, protein–protein interactions are impacted by lactylation. Membrane-organizing extension spike protein (MOESIN) K72la forms hydrogen bonds with the transforming growth factor-β (TGF-β) type I receptor, strengthening their interaction (Gu et al., 2022) (Figure 2). In gastric cancer research, Nijmegen breakage syndrome 1 (NBS1) K388la promotes binding to MRE11, enabling MRE11-RAD50-NBS1 complex formation and DNA damage repair (Chen H. et al., 2024). α-MHC K1897la enhances its interaction with titin; in contrast, the K1897R mutation, which prevents lactylation, considerably reduces this interaction (Zhang N. et al., 2023). X-ray repair cross-complementing protein 1 (XRCC1) K247la increases the interaction with importin α in glioblastoma stem cells (GSCs) (Li G. et al., 2024). Vps34 lactylation at K356/K781 reinforces its interactions with Beclin 1, autophagy-related protein 14-like protein, and UV radiation resistance-associated gene, promoting lipid kinase activity (Sun W. et al., 2023; Jia et al., 2023).

Moreover, lactation can inhibit protein interactions. For instance, transcription factor EB (TFEB) K91la inhibits the interaction with WW domain containing E3 ubiquitin protein ligase 2 (WWP2) in pancreatic cancer cells (Huang Y. et al., 2024) (Figure 2). Compared with nonlactylated p53, 100-fold, 10-fold, and 1,000-fold reductions in the binding affinities of p53 K120la, p53 K139la, and p53 K120/K139la were observed (Zong et al., 2024). Aldolase A (ALDOA) K230/K322la weakens the binding to DEAD-box helicase 17 in liver cancer stem cells (Feng et al., 2024), and Ikzf1 K164la significantly decreases the binding to TH17 differentiation-related gene promoters (Fan W. et al., 2023).

Polypyrimidine tract-binding protein 1 (PTBP1) K436la displays bidirectional regulatory effects, disrupting hydrogen bond formation with TRIM21 while augmenting its binding to the 3′UTR of 6-phosphofructo-2-kinase/fructose-2,6-biphosphatase four mRNA (Figure 2). The effect of lactylation on all molecular interactions of a protein is not likely to be uniform owing to the selective modulation. This process specifically alters binding interfaces via conformational changes (Zhou et al., 2025).

The bidirectional regulatory effects of lactylation on nonhistone molecular interactions imply that its influence on protein function may be highly specific. Its underlying mechanisms and pathological significance warrant further exploration.

### 3.2 Protein stability

Lactylation considerably affects nonhistone protein stability. Several studies have reported that the stability of proteins such as lymphocyte cytosolic protein 1, hypoxia-inducible factor-1α (HIF-1α), carcinoembryonic antigen-related cell adhesion molecule 6 (CEACAM6), β-catenin, nucleolar and spindle associated protein 1 (NUSAP1), and phosphofructokinase P (PFKP) is enhanced after lactylation (Zhang W. et al., 2023; Luo et al., 2022; Chu et al., 2023; Miao et al., 2023; Chen et al., 2023; Mi, 2024) and is therefore positively regulated. When glycolysis is inhibited, the lactylation levels of these proteins are reduced, which decreases their stability, revealing a direct link between lactylation and cellular metabolic status.

One of the primary mechanisms by which lactylation influences nonhistone stability is by antagonizing the ubiquitination pathway. Fan identified K70 as the sole lactylation site of apolipoprotein C2 (APOC2). Together with K52, K61, and K96 residues, it serves as the target for ubiquitination (Chen J. et al., 2024). Lactate treatment substantially reduced the ubiquitination of wild-type APOC2 but did not affect the lactylation-deficient APOC2-K70R mutant. These findings confirmed that K70 lactylation maintains APOC2 stability by inhibiting ubiquitination (Chen J. et al., 2024) (Figure 2). Similar mechanisms operate in other proteins too. For instance, TFEB K91la disrupts its interaction with E3 ubiquitin ligase WWP2, lowering TFEB ubiquitination and proteasomal degradation in pancreatic cancer (Huang Y. et al., 2024). In glioma stem cells, PTBP1 K436la disrupts the formation of hydrogen bonds with the E3 ubiquitin ligase TRIM21, preventing proteasomal degradation and improving stability (Zhou et al., 2025) (Figure 2). In cervical cancer, discoidin, CUB and LCCL domain-containing 1 (DCBLD1) K172la inhibits ubiquitination, enhancing the stability and extending the protein half-life (Meng et al., 2024).

Besides regulating the ubiquitin–proteasome degradation pathway, lactylation controls protein stability via other mechanisms. In colorectal cancer (CRC), Tong et al. found that programmed death-ligand 1 (PD-L1) lactylation inhibits its degradation via the lysosomal pathway. Consequently, PD-L1 protein expression is increased, and PD-L1 mRNA is maintained at a constant level, revealing a novel lactylation mechanism that regulates the lysosomal degradation pathway (Tong et al., 2024). Furthermore, K147-lactylated ALDOA shows better thermal stability than the wild-type enzyme, signifying that lactylation directly affects the physicochemical properties of proteins (Shao et al., 2025).

In conclusion, lactylation regulates nonhistone protein stability by antagonizing ubiquitination, enhancing thermal stability, and inhibiting lysosomal degradation. These processes involve complex molecular interactions and crosstalk among various PTMs, constituting a complex regulatory network that modulates protein stability and influences cellular functions in physiological and pathological contexts.

### 3.3 Enzymatic activity

Lactylation of nonhistone proteins exerts bidirectional regulatory effects on enzymatic activity.

Many studies have asserted the ability of lactylation to enhance the catalytic activity of enzymes. Nicotinamide mononucleotide adenylyltransferase 1 (NMNAT1) K128la is essential for maintaining enzymatic activity and nuclear NAD^+^ levels. The K128R delactylation mutant exhibits significantly reduced activity than the wild-type enzyme (Huang H. et al., 2024). In CRC, eukaryotic translation elongation factor 1 alpha 2 (eEF1A2) K408la improves GTPase activity in response to aa-tRNA stimulation, accelerating ribosomal translation elongation and promoting cancer cell proliferation (Xie et al., 2024). NOP2/Sun RNA methyltransferase 2 (NSUN2) K508la augments its RNA methyltransferase catalytic activity (Niu et al., 2025). In gastric cancer, METTL16 K229la status is directly correlated with its enzymatic activity. The K229R delactylation mutant shows decreased methyltransferase activity, whereas the K229E lactylation-mimicking mutant exhibits substantially improved activity (Sun L. et al., 2023). Moreover, pyruvate kinase M2 (PKM2) K62la contracts the amino acid binding pocket via conformational changes, stabilizing the tetrameric conformation and promoting the catalytic activity (Wang J. et al., 2022) (Figure 2).

Conversely, lactylation inhibits the catalytic activity of some enzymes. For instance, ALDOA K147la considerably reduces the enzymatic activity compared with the wild-type (Wan et al., 2022) (Figure 2). In 2025, researchers successfully introduced a precise K147la modification into ALDOA in HEK293T cells using the genetic code expansion technology. Functional analyses verified that lactylation significantly inhibited the enzymatic activity *in vivo* (Shao et al., 2025). Hypoxic conditions induced the accumulation of AARS2, which catalyzed PDHA1 K336la and CPT2 K457/K458la. Lactylation inactivated both enzymes and inhibited mitochondrial oxidative phosphorylation (Mao et al., 2024). Adenylate kinase 2 K28la substantially decreases the enzymatic activity and is correlated with hepatocellular carcinoma progression (Yang Z. et al., 2023).

### 3.4 Subcellular localization

Advanced LC–MS/MS technologies have aided in systematically identifying and analyzing nonhistone lactylation sites and their subcellular distribution. In gastrointestinal tumors, lactylation sites are distributed across the cytoplasm (39.58%), nucleus (35.41%), mitochondria (7.64%), extracellular regions, cell membrane, and endoplasmic reticulum (Chen J. et al., 2024). However, related research reported slightly different findings, with the highest proportion in the nucleus, followed by the cytoplasm, mitochondria, extracellular regions, and cytoplasmic membrane (Duan et al., 2024). The extensive distribution of lactylation implies that it may regulate protein function via subcellular localization, providing essential insights into cellular physiological and pathological processes.

Several studies have confirmed the role of lactylation in promoting the nuclear localization of proteins. ABCF1 K430la, which is upregulated in hepatocellular carcinoma, alters the protein conformation and exposes the nuclear localization sequence (NLS), guiding ABCF1 into the nucleus (Figure 2) and activating the HIF1 signaling pathway (Hong et al., 2025). In addition, lactate treatment augments Snail1 lactylation, increasing its nuclear translocation and TGF-β gene binding (Fan M. et al., 2023). NMNAT1 K128la, present within the NLS (K123-K129), considerably improves its nuclear localization (Huang H. et al., 2024). In GSCs, XRCC1-K247 lactylation aids its nuclear translocation and enhances DNA damage repair (Li G. et al., 2024). ALDOA K147la stimulates cytoplasm-to-nucleus translocation (Shao et al., 2025). Moreover, the oncogene c-Myc exhibits elevated nuclear localization and heightened protein levels under high lactate conditions, an effect eliminated by the P300 inhibitor C464 (Li Y. et al., 2024). Under high lactate conditions, increased lactylated and total c-Myc protein levels are seen in the nucleus. Upon inhibiting lactylation with the P300 inhibitor C464, the previously noted increase in nuclear c-Myc was eliminated (Li Y. et al., 2024). Transcription factor Twist1-K150 lactylation (rather than K73/K76) stimulates its nuclear translocation (Xu Y. et al., 2024).

Nonetheless, the effects of lactylation on nonhistone localization are not unidirectional. Wang observed that p300-mediated PKM2 lactylation can suppress its nuclear translocation (Wang et al., 2024). Similarly, another research uncovered that the PKM2-K62R mutant shows approximately 1.41-fold higher nuclear levels than the wild-type, which confirms that K62 lactylation inhibits nuclear localization when exogenous lactate is added (Wang J. et al., 2022). Lactylation in proximity to the NLS of high-mobility group box 1 (HMGB1) augments its cytoplasmic translocation (Yang et al., 2022) (Figure 2). In addition, mitochondrial elongation factor Tu (TUFM) K286la prevents its mitochondrial translocation by suppressing its interaction with the translocase of the outer mitochondrial membrane 40 (Weng et al., 2025) (Figure 2).

As proteins perform specific roles in various cellular compartments, regulating subcellular localization is a key mechanism by which lactylation affects protein function. This regulatory mechanism offers a new perspective on the functional significance of lactylation.

A comprehensive review of studies reveals that lactylation regulates nonhistone functions via multiple coordinated mechanisms rather than a single mechanism. ALDOA exemplifies this comprehensive regulation, with lactylation simultaneously altering its molecular interaction network, protein stability, enzymatic activity, and subcellular localization, resulting in functional reshaping (Shao et al., 2025).

## 4 The impact of nonhistone lactylation on the biological functions of cancer cells

**FIGURE 3:**
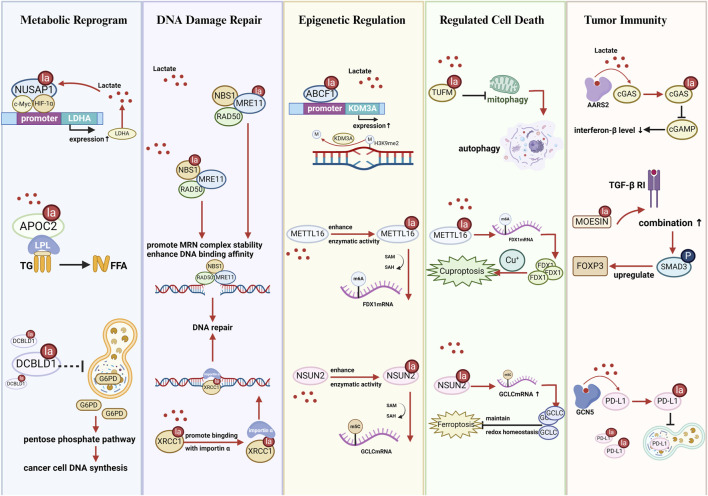
Schematic illustration of nonhistone lactylation affects the biological functions of cancer cells. *Metabolic reprogramming*: Kla regulates glycolysis, lipid metabolism, amino acid metabolism, and the pentose phosphate pathway by altering enzyme activity, stability, or expression. *DNA damage repair*: Kla enhances the activity and nuclear localization of DNA repair proteins (e.g., MRE11, NBS1, XRCC1), promoting homologous recombination and resistance to chemo/radiotherapy. *Epigenetic regulation*: Kla affects RNA methylation (m6A, m5C), histone modification, and transcriptional programs by modifying RNA-binding proteins or chromatin remodelers (e.g., NSUN2, IGF2BP3, METTL16, ABCF1). *Regulated cell death*: Kla modulates apoptosis, autophagy, ferroptosis, and cuproptosis by influencing metabolic enzymes and mitochondrial dynamics (e.g., SIRT3, PFKP, TUFM, HMGB1). *Tumor immunity*: Kla shapes the tumor immune microenvironment by regulating immune-related proteins and immune cell polarization (e.g., PD-L1, MOESIN, METTL3, APOC2, FOXP3+ cells), promoting immune evasion and therapy resistance. Abbreviations NUSAP1: nucleolar and spindle associated protein 1; LDHA: lactate dehydrogenase A; LPL: lipoprotein lipase; TG: triglyceride; FFA: free fatty acid; DCBLD1: discoidin, CUB and LCCL domain containing 1; G6PD: glucose-6-phosphate dehydrogenase; NBS1: Nijmegen breakage syndrome 1; MRE11: meiotic recombination 11; XRCC1: X-ray repair cross-complementing protein 1; ABCF1: ATP-binding cassette transporter F1; KDM3A: lysine demethylase 3A; METTL16: methyltransferase-like protein 16; FDX1: ferredoxin 1; NSUN2: NOP2/Sun RNA methyltransferase 2; GCLC: glutamate-cysteine ligase catalytic subunit; TUFM: mitochondrial elongation factor Tu; AARS2: alanine-tRNA synthetase 2; cGAS: cyclic GMP-AMP synthase; cGAMP: cyclic GMP-AMP; MOESIN: Membrane-organizing extension spike protein; TGF-β RI: transforming growth factor-β type I receptor; FOXP3: forkhead box P3; GCN5: general control non-derepressible 5; PD-L1: programmed death-ligand 1.

Nonhistone lactylation represents an emerging PTM that plays crucial roles in cancer biology by dynamically regulating multiple cellular processes. This modification influences cancer cell biology through several mechanisms, including metabolic reprogramming, DNA damage repair, epigenetic modulation, cell death pathways, and tumor immunity (Figure 3).

### 4.1 Metabolic Reprograming

Enrichment analyses have demonstrated that differentially expressed proteins with lactylation modifications are significantly enriched in metabolic pathways such as glycolysis, fatty acid metabolism, and amino acid metabolism, strongly influencing tumor initiation and progression (Gaffney et al., 2020; Yang Z. et al., 2023; Duan et al., 2024; Wang J. et al., 2022). Cancer cells undergo metabolic reprogramming to obtain the substantial amount of energy required for their rapid proliferation. This reprogramming upregulates glycolysis, increases lactate production and accumulation, and further promotes lactylation modification (He et al., 2024). Metabolism-linked nonhistone lactylation regulates glycolysis by altering protein function, creating glycolysis–lactylation–a glycolysis feedback loop. The following examples further illustrate how nonhistone lactylation contributes to metabolism-driven feedback loops in various cancer types.

In hepatocellular carcinoma cell line (HepG2), ABCF1 K430la facilitates tumor progression by stimulating the expression of HIF1A and its downstream molecules, enhancing lactate production. Increased lactate levels further promote ABCF1-K430 lactylation, establishing a positive feedback loop of lactate–ABCF1-HIF1A–lactate (Hong et al., 2025). In CRC cell lines (SW620 and RRKO), lactate produced via glycolysis promotes β-catenin lactylation, augmenting its stability. β-catenin knockdown inhibits glycolysis indicators such as lactate production and extracellular acidification rate, confirming the glycolysis–β-catenin-Kla–glycolysis positive feedback loop (Miao et al., 2023). In pancreatic cancer, NUSAP1 lactylation increases its stability and forms a complex with c-Myc and HIF-1α, binding to the LDHA promoter region. This binding promotes LDHA expression and glycolysis, establishing lactate–NUSAP1-Kla-LDHA–lactate positive feedback pathway (Chen et al., 2023) (Figure 3). In ovarian cancer, lactate accumulation stimulates phosphofructokinase P (PFKP) K392la, suppressing PTEN expression and enhancing glycolysis (Mi, 2024). Nevertheless, in HEK293T cells, ALDOA K147la abrogates enzymatic activity and inhibits glycolytic flux, creating a negative feedback regulation (Shao et al., 2025). Thus, the feedback loop mechanism between nonhistone lactylation and glycolysis must be investigated further to decipher its regulatory roles across different cell types and pathological conditions.

Nonhistone lactylation also regulates metabolic pathways other than glycolysis. Oxidative phosphorylation is negatively regulated by mitochondrial protein lactylation. Under hypoxic conditions, AARS2 accumulation promotes PDHA and CPT2 lactylation, preventing their enzymatic activities and curbing acetyl-CoA production, which shifts the metabolism toward glycolysis (Mao et al., 2024). Lactylome analysis of non-small cell lung cancer revealed that APOC2 is the only upregulated lactylated protein involved in lipid transport and metabolism. APOC2 K70la improves extracellular lipolysis and promotes free fatty acid release (Chen J. et al., 2024) (Figure 3). PCK2 K100la enhances enzymatic activity and competitively inhibits ubiquitin-mediated degradation of mitochondrial 3-oxoacyl-ACP synthase (OXSM), enabling the metabolic remodeling of mitochondrial fatty acid synthesis (Yuan et al., 2025).

IGF2BP3 K76la upregulates PCK2 expression in lenvatinib-resistant hepatocellular carcinoma cells, triggering serine metabolism reprogramming and conferring drug resistance (Lu et al., 2024). DCBLD1 K172la suppresses its ubiquitination, stabilizes the protein, and extends its half-life. By inhibiting the autophagic degradation of glucose-6-phosphate dehydrogenase, DCBLD1 K172la activates the pentose phosphate pathway, providing crucial precursors for nucleotide biosynthesis in cancer cells (Meng et al., 2024) (Figure 3).

### 4.2 DNA damage and repair

The MRE11-RAD50-NBS1 complex is critical for sensing and repairing DNA double-strand breaks (DSBs). Chen et al. identified that MRE11 K673la increases its DNA-binding affinity, promoting homologous recombination repair and leading to chemotherapeutic resistance in basal-like breast cancer (Chen Y. et al., 2024). Similarly, NBS1 lactylation at K388 considerably improves MRE11-RAD50-NBS1 complex stability at DSB sites, enhancing homologous recombination repair efficiency (Chen H. et al., 2024). In GSCs, lactylation of XRCC1 at K247 promotes its nuclear translocation, mediating DNA damage repair and conferring resistance to radiotherapy and chemotherapy (Li G. et al., 2024) (Figure 3).

### 4.3 Epigenetic regulation

Nonhistone lactylation impacts epigenetic processes via multiple pathways, creating a complex regulatory network.

NSUN2 K508la boosts its RNA methyltransferase activity, stimulating 5-methylcytosine modification of the glutamate–cysteine ligase catalytic subunit (GCLC) mRNA and affecting the cellular redox balance (Niu et al., 2025). In lenvatinib-resistant hepatocellular carcinoma, lactylation of IGF2BP3 at K76 promotes the generation of S-adenosylmethionine and induces RNA m^6^A modification, leading to drug resistance (Lu et al., 2024). In gastric cancer, METTL16 K229la increases m^6^A modification of ferredoxin 1 mRNA, which triggers cuproptosis (Sun L. et al., 2023). Furthermore, nonhistone lactylation regulates histone PTMs. ABCF1 K430la mediates its nuclear translocation, upregulates KDM3A expression, and enhances H3K9me2 demethylation (Figure 3), activating the HIF1A pathway to promote hepatocellular carcinoma progression (Hong et al., 2025). In addition, histone lactylation itself is a unique epigenetic modification (Zhang et al., 2019). Lactylation of nonhistones is predominantly enriched in metabolic enzyme pathways (Yang Z. et al., 2023), signifying that any enzyme affecting glycolysis could theoretically influence lactylation modifications, including histone lactylation.

These mechanisms indicate complex interactions between nonhistone lactylation and epigenetic modifications, providing key information on metabolic–epigenetic crosstalk and potential cancer therapeutic targets.

### 4.4 Regulated cell death

Nonhistone lactylation regulates numerous cell death pathways such as ferroptosis, cuproptosis, apoptosis, and autophagy (Zhao et al., 2020).

Nonhistone lactylation exerts bidirectional effects on ferroptosis regulation. NSUN2 K508la augments glutathione synthesis, suppressing ferroptosis in gastric cancer cells (Niu et al., 2025). This cascade reaction increases GSH synthesis, maintains redox homeostasis, effectively suppresses lipid peroxidation, and ultimately suppresses ferroptosis (Figure 3). Conversely, Yuan et al. reported that PCK2 K100la stabilizes OXSM, promotes mitochondrial fatty acid synthesis, and activates hepatocyte ferroptosis (Yuan et al., 2025). METTL16 K229 upregulates ferredoxin 1 in gastric cancer, disrupting copper metabolism and inducing cuproptosis (Sun L. et al., 2023) (Figure 3).

In addition, nonhistone lactylation affects apoptosis and autophagy. SIRT3, a lactylation eraser, induces apoptosis in hepatocellular carcinoma by inhibiting cyclin E2 lactylation (Jin et al., 2023). Adenylate kinase 2 K28la prevents its kinase activity, downregulating apoptotic pathways (Yang Z. et al., 2023). Weng et al. showed that TUFM K286la inhibits its interaction with the outer mitochondrial membrane complex, impairing mitophagy and driving cells toward apoptosis (Weng et al., 2025) (Figure 3). Lactylation of HMGB1 near its NLS facilitates cytoplasmic translocation, improving macrophage secretion of HMGB1 (Yang et al., 2022) and activating autophagy via the advanced glycosylation end-product-specific receptor or toll-like receptor (Chen R. et al., 2024; Kim et al., 2021). Moreover, PFKP lactylation inhibits PTEN activity (Mi, 2024) and indirectly suppresses autophagy (Zhang K. K. et al., 2023).

The regulation of programmed cell death by nonhistone lactylation is complex and context-dependent, differing based on the tumor type, metabolic status, microenvironment, and signaling pathways. Hence, further studies should focus on mechanistic investigations and functional validations across various cancer types and experimental models.

### 4.5 Tumor immunity

Nonhistone lactylation critically shapes the tumor microenvironment by modulating key immune-related proteins.

In innate immunity, AARS2-mediated lactylation of cGAS results in its inactivation, decreasing cGAMP and interferon-β levels and weakening immune responses (Li H. et al., 2024). MOESIN lactylation activates SMAD3 signaling, upregulates forkhead box P3 (FOXP3), and stimulates the differentiation of regulatory T cell, leading to immunosuppression (Gu et al., 2022). Ikzf1 K164la promotes TH17 cell differentiation by increasing the binding to Runx1, Tlr4, IL2, and IL4 promoters (Fan W. et al., 2023).

Macrophage polarization plays a crucial role in the tumor microenvironment. Proinflammatory macrophages activate T cells and natural killer cells, disrupting tissue integrity and impeding tumor progression. Conversely, reparative macrophages are involved in anti-inflammatory responses and tissue remodeling, fostering tumor growth, invasion, and immune suppression (Locati et al., 2020). PKM2 K62la triggers macrophage transition from the proinflammatory to the reparative phenotype, facilitating tumor immune evasion (Wang J. et al., 2022). Innon-small cell lung cancer, APOC2 K70la augments its stability, increasing free fatty acid release and Treg accumulation (Chen J. et al., 2024). In CRC, lactate induces H3K18 lactylation in tumor-infiltrating macrophages, leading to the upregulation of METTL3. Moreover, it directly induces METTL3 K281/K345 lactylation within the target recognition domain. This lactylation activates the METTL3-m6A-JAK1-STAT3 axis, leading to myeloid cell immunosuppression (Xiong et al., 2022). PD-L1 K810–813 lactylations retard its degradation, maintaining its high expression and inhibiting T-cell activation, proliferation, and cytotoxicity (Tong et al., 2024). HMGB1 lactylation near its NLS stimulates cytoplasmic translocation and exosomal secretion, with extracellular HMGB1 activating HIF1A via advanced glycosylation end-product-specific receptor, upregulating PD-L1, and triggering immunosuppression (Yang et al., 2022) (Figure 3). Furthermore, FOXP3^+^ natural killer T-like cells sustain the immunosuppressive function in malignant pleural effusion by ensuring high lactylation levels (Wang Z.-H. et al., 2023).

Nonhistone lactylation exerts multifaceted effects on the tumor immunosuppressive microenvironment by regulating immune cell differentiation, function, and phenotype. Previous studies noted that lactate inhibits several immune cells, including dendritic cells, T cells, and natural killer cells. However, whether these effects occur via nonhistone lactylation requires further investigation (Fischer et al., 2007; Certo et al., 2021). In addition, future research should focus on the specific roles of lactylation across different immune cells, providing a robust theoretical foundation for developing more effective antitumor immunotherapies.

## 5 High levels of nonhistone lactylation and its association with tumor progression, metastasis, and poor prognosis

Nonhistone lactylation is intricately linked to tumor initiation, progression, and prognosis (Chen et al., 2025a; Dai et al., 2024).

KEGG and Hallmark enrichment analyses in gastric cancer revealed strong correlations between lactylation scores and oncogenic pathways such as WNT, TGF-β, mTOR, and P53 signaling (Yang H. et al., 2023). Lactylome analysis of 40 gastrointestinal tumor samples indicated considerably higher lactylation levels in tumor tissues than in normal adjacent tissues. Moreover, increased lactylation was correlated with greater invasiveness and poorer clinical outcomes (Duan et al., 2024). In HBV-related hepatocellular carcinoma, the expression of the lactylation eraser SIRT3 was substantially lower in tumor tissues and was negatively correlated with the tumor stage (Gao et al., 2019).

The lactylation of several key proteins significantly affects tumor proliferation and metastasis. Adenylate kinase 2 K28la reduces its activity, stimulating hepatocellular carcinoma proliferation and metastasis (Yang Z. et al., 2023). AARS1 catalyzes p53 lactylation (K120 and K139) within its DNA-binding domain, disrupting its function and accelerating tumorigenesis (Zong et al., 2024). CBX3 K10la improves the binding to H3K9me3 in gastrointestinal tumors, enhancing tumor invasiveness (Duan et al., 2024). In cervical cancer, elevated lactate levels in tumor tissues promote HIF-1α enrichment at the DCBLD1 promoter region, augmenting DCBLD1 mRNA expression. DCBLD1 lactylation activates the pentose phosphate pathway, promoting proliferation and invasion (Meng et al., 2024). In CRC, KAT8-mediated eEF1A2 K408la improves translational efficiency and enhances cancer cell proliferation (Xie et al., 2024). AARS1 and YAP-TEAD1 constitute a positive feedback loop in which AARS1 catalyzes YAP-TEAD1 lactylation while YAP-TEAD1 upregulates AARS1 expression, leading to gastric cancer cell proliferation and apoptosis suppression (Ju et al., 2024).

Furthermore, nonhistone lactylation considerably affects cancer stem cells maintenance. METTL3 lactylation creates a lactylation-m6A-JAK1-STAT3 axis in CRC, causing immunosuppression and promoting tumor progression (Xiong et al., 2022). Nucleolin K477la activates the MADD/ERK pathway in cholangiocarcinoma and accelerates tumor growth in xenograft models (Yang et al., 2024). In hepatocellular carcinoma, SIRT3 reduction promotes cyclin E2 lactylation, enhancing the proliferation and invasion of hepatocellular carcinoma cells (Jin et al., 2023). In liver cancer, ABCF1 K430la activates the KDM3A-H3K9me2-HIF1A axis, with K430Q mutants displaying greater tumor growth and metastasis in mouse models (Hong et al., 2025). In gallbladder cancer, YY1 K183la increases F-box protein 33 (FBXO33) transcription, regulating the FBXO33-p53 axis, promoting epithelial–mesenchymal transition, and inducing tumor invasion (Wu et al., 2025). In prostate cancer, lactylation stabilizes HIF-1α, activating KIAA1199 transcription and stimulating angiogenesis and tumor metastasis (Luo et al., 2022).

Maintaining cancer stem cells s is an important mechanism that drives tumor progression and therapeutic resistance. In hepatocellular carcinoma, ALDOA K230/K322 lactylation weakens its strong binding affinity with DEAD-box helicase 17, leading to their dissociation in the cytoplasm and promoting DEAD-box helicase 17 nuclear translocation and SOX2 upregulation, sustaining liver cancer stem cells properties (Feng et al., 2024). PTBP1 K436la enhances GSC proliferation and stemness (Zhou et al., 2025). β-catenin lactylation activates the Wnt pathway in CRC, enhancing the proliferation and stemness of cancer cells (Miao et al., 2023). However, not all lactylation modifications promote tumor progression. In gastric cancer, increased copper level improves AARS1/AARS2-METTL16 interaction. METTL16K229la mediates the m6A methylation of ferredoxin 1 mRNA, triggering cuproptosis (Sun L. et al., 2023).

These findings highlight the functions of nonhistone lactylation in promoting cancer progression and metastasis while occasionally inducing cell death under specific conditions. Further studies on these mechanisms may provide novel therapeutic targets for cancer.

## 6 Nonhistone lactylation and resistance to antitumor therapy

Multiple studies have proved that nonhistone lactylation significantly influences tumor sensitivity to various therapeutic approaches (Table 2).

**TABLE 2:** Nonhistone lactylation and resistance to antitumor therapy.

Therapy type	Molecular target and Kla site	Associated mechanism	Experimental model	Therapeutic implications
Chemo-therapy	CEACAM6; not mentioned (Chu et al., 2023)	ALDOB drives lactagenesis and promotes CEACAM6 Kla → enhancing CEACAM6 stabilization → 5-FU chemotherapy resistance	*In vitro*: HT29 and WiDr cell lines *In vivo*: BALB/c nude mice xenograft tumor model	Targeting CEACAM6 Kla for 5-fluorouracil chemosensitization; disrupting the ALDOB/lactate/CEACAM6 axis combined with standard chemotherapy may improve treatment efficacy
	p53; K120/K139 (Zong et al., 2024)	Aerobic glycolysis increases lactate production → activateed AARS1 catalyze p53 Kla → p53 inactivation facilitates doxorubicin resistance	*In vitro*: HEK293T, HeLa, p53+/+ and p53−/− HCT116 cell lines *In vivo*: BALB/c nude mice xenograft tumor model	Chemothrapy resistance can be reversed by β-alanine competitive inhibition
	MRE11; K673 (Chen et al., 2024a)	Improved DNA end resection and homologous recombination repair	*In vitro*: patient-derived organoids	Resistance to PARP inhibitors, olaparib, cisplatin can be reversed by CBP/LDH inhibition
Radio-therapy	XRCC1; K247 (Li et al., 2024b)	ALDH1A3-PKM2 Interaction promotes PKM2 tetramer formation → enhancing glycolytic activity → XRCC1 Kla promotes DNA damage repair	*In vitro*: BNI-20-1-S, BNI-21-1-S and N33 cell lines Patient-derived glioblastoma organoids	Radiotherapeutic and temozolomide resistance can be reversed by D34-919 (molecule inhibitor of ALDH1A3-PKM2 interaction)
Immuno-therapy	APOC2; K70 (Chen et al., 2024c; Kumagai et al., 2020)	Lactate enhances APOC2 Kla via p300 → enhancing APOC2 stabilization → upregulates Extracellular lipolysis and FFA release → Treg accumulation, CD8^+^ T cell reduction and immunosuppression	*In vivo*: H1299 and LLC cell lines	Immunotherapeutic resistance overcome by anti-APOC2-K70lac antibody or APOC2-K70R mutation interference or FX11 + anti-PD-1
	MOESIN; K72 (Gu et al., 2022)	MOESIN Kla strengthens interaction with TGF-β type I receptor → activate SMAD3pathway → Increase FOXP3 expression and promote the generation of Treg cells → leading to immunosuppressive microenvironment	*In vivo*: Hepa1-6 cell lines	Cotreatment with anti-PD-1 and a lactate dehydrogenase inhibitor has a stronger antitumor effect than anti-PD-1 (camrelizumab) alone
Targeted therapy	IGF2BP3; K76 (Lu et al., 2024)	IGF2BP3 Kla stabilizes PCK2 and NRF2 mRNAs through m6A modification → serine metabolism reprogramming, enhanced antioxidant ability → promoting lenvatinib resistance in HCC.	*In vivo*: Hep3B-LR, Huh7-LR and Hepa1-6-LR (Lenvatinib-resistant cell lines) *In vivo*: BALB/c nude mice xenograft tumor model	Inhibition of IGF2BP3 Kla can disrupt the IGF2BP3-PCK2-SAM-m6A axis, restoring lenvatinib sensitivity Utilizing glycolysis inhibitors 2-DG may reduce IGF2BP3 Kla and enhance the efficacy of antitumor therapies

### 6.1 Chemotherapeutic resistance

CEACAM6 is highly expressed in malignant tumors and results in resistance to multiple chemotherapeutic agents (Duxbury et al., 2004; Rizeq et al., 2018). Recent findings suggest that CEACAM6 lactylation improves its stability, modulates cancer cell sensitivity to 5-fluorouracil, and induces chemotherapeutic resistance in CRC cell lines (HT29 and WiDr) (Chu et al., 2023). However, this conclusion is based on a single study with limited sample size and lacks validation in primary tumor samples or clinical cohorts. AARS1-catalyzed p53 lactylation facilitates doxorubicin resistance, which can be reversed by β-alanine via competitive inhibition of lactate binding (Zong et al., 2024). In basal-like breast cancer, MRE11 K673la improves DNA end resection and homologous recombination repair, conferring resistance to poly ADP-ribose polymerase inhibitors, olaparib, and cisplatin (Chen Y. et al., 2024). In patient-derived organoid models, effectively inhibiting CBP and LDH reverses this resistance. Researchers have designed K673-pe, a peptide inhibitor that specifically blocks MRE11 lactylation, augmenting the antitumor therapeutic response (Chen Y. et al., 2024). NSUN2 K508la upregulates glutathione levels in gastric cancer, enhancing the resistance to ferroptosis-inducing agents such as doxorubicin and RSL3. NSUN2 knockout downregulates GCLC expression and GSH synthesis, thereby increasing chemotherapeutic sensitivity (Niu et al., 2025).

### 6.2 Radiotherapeutic resistance

Exogenous lactate treatment alleviates p53-mediated cell death in irradiated mice, with p53 lactylation at K120/K139 impairing its DNA-binding ability and mediating radiotherapeutic resistance (Zong et al., 2024). Li et al. found that aldehyde dehydrogenase 1 A3 (ALDH1A3) overexpression in glioblastoma promotes tetrameric PKM2 formation, increasing XRCC1 K247 lactylation. This process enhances DNA damage repair and protein stability, leading to radiotherapeutic and temozolomide resistance (Li G. et al., 2024). The compound D34-919 disrupts ALDH1A3-PKM2 interaction and decreases lactylation, restoring glioblastoma sensitivity to radiotherapy (Li G. et al., 2024). Developing small molecules targeting XRCC1 K247 lactylation may similarly augment the efficacy of radiotherapy and chemotherapy. Moreover, HMGB1 lactylation stimulates its extracellular release (Yang et al., 2022), and earlier studies have asserted that the release of HMGB1 could attenuate radiotherapeutic efficacy (Shinde-Jadhav et al., 2021).

### 6.3 Immunotherapeutic resistance

Nonhistone lactylation shapes the tumor immune microenvironment, influencing immunotherapeutic sensitivity. In non-small cell lung cancer, APOC2 K70 lactylation is substantially upregulated in patients resistant to immunotherapy and is negatively linked to overall survival in both non-small cell lung cancer and gastric cancer (Chen J. et al., 2024). APOC2 K70 lactylation enhances Treg metabolism and reduces CD8^+^ T cell frequency (Kumagai et al., 2020). Anti-APOC2-K70lac antibody or LDH inhibitor FX11, combined with anti-PD-1 therapy, substantially decreases Treg frequency and the proportions of TNF-α^+^ IFN-γ^+^ and CD69^+^ CD8^+^ T cells in the tumor microenvironment, conferring immunotherapeutic resistance (Chen J. et al., 2024). In hepatocellular carcinoma, lower MOESIN lactylation is associated with improved response to camrelizumab, implying that patients with lower lactylation levels benefit more from anti-PD-1 therapy (Gu et al., 2022). Lactylation scoring models in gastric cancer and hepatocellular carcinoma indicate that patients with high lactylation scores display stronger immune evasion and lower immunotherapeutic response rates (Yang H. et al., 2023; Cheng et al., 2023).

### 6.4 Targeted therapy resistance

In lenvatinib-resistant hepatocellular carcinoma, proteomic analysis showed elevated lactylation modifications, especially IGF2BP3 K76la, which enhances serine metabolism reprogramming and m6A modification, improving antioxidant ability and lenvatinib resistance (Lu et al., 2024). Not all studies indicate a direct correlation between reduced lactylation and increased targeted therapy sensitivity. For instance, a bioinformatics-based lactylation scoring model revealed that while most drugs exhibited higher sensitivity in the low-lactylation score group, gefitinib and metoprolol were more effective in the high-lactylation score group (Yang H. et al., 2023). Similarly, another investigation identified that patients with high lactylation scores were more sensitive to sorafenib than those with low scores (Cheng et al., 2023). These diverse findings suggest that the effect of lactylation on drug sensitivity may vary considerably depending on the tumor type, therapeutic modality, and molecular background. Hence, specific lactylation sites and their functional mechanisms should be examined to develop precise therapeutic strategies.

It is important to note that current lactylation research faces several methodological limitations. Most studies rely on cell line models rather than primary tissues, and sample sizes are limited. Future studies should prioritize validation in clinical samples and larger cohorts.

## 7 Targeting nonhistone lactylation modifications for antitumor therapy

The above findings highlight a novel strategy to enhance the therapeutic efficacy for patients with tumors: target lactylation modification to act as an antitumor treatment.

Cancer cells generate high levels of lactate via aerobic glycolysis, leading to lactate accumulation in both cancer cells and the tumor microenvironment. Therefore, inhibiting lactate production and transport represents a promising strategy to modulate lactylation modifications (Sharma et al., 2022).

The GLUT1 inhibitor BAY876 decreases glucose uptake, suppressing glycolysis and reducing lactate levels in the tumor microenvironment. Preclinical studies demonstrate that BAY876 enhances immunotherapeutic efficacy in glioblastoma (Li T. et al., 2024). In xenograft and glioblastoma organoid models, D34-919 blocks the ALDH1A3-PKM2 interaction, inhibiting XRCC1 lactylation and overcoming temozolomide and radiotherapeutic resistance in GSCs (Li G. et al., 2024).

LDHA, highly expressed in tumors, catalyzes the conversion of pyruvate to lactate. The LDHA inhibitor stiripentol reduces DNA repair efficiency by inhibiting NBS1 K388la. Therefore, this receptor exhibits synergistic effects with cisplatin or radiotherapy in patient-derived organoid and xenograft models (Chen H. et al., 2024). Oxamate increases apoptosis and autophagy in non-small cell lung cancer cells when combined with radiotherapy, enhancing radiotherapeutic sensitivity (Yang et al., 2021). Several other LDHA inhibitors, such as AT-101 and FX-11, though not yet in clinical practice, hold immense promise for inhibiting lactate production in antitumor clinical treatment (Doherty and Cleveland, 2013; Le et al., 2010; Wu et al., 2021; Lv et al., 2023).

Effective inhibition of lactate transport can reduce its accumulation. AZD3965, a selective monocarboxylate transporter (MCT) 1/2 inhibitor in phase I/II clinical trials, enhances the efficacy of immune checkpoint inhibitors, particularly in lymphoma and non-small cell lung cancer (Barnes et al., 2020; Lopez et al., 2023; Beloueche-Babari et al., 2020; Noble et al., 2017; Halford et al., 2023; Guan and Morris, 2020; Apicella et al., 2018). The 7ACC series compounds inhibit both MCT1 and MCT4, blocking lactate transport mechanisms and exhibiting synergistic effects with radiotherapy in a xenograft model (Draoui et al., 2014; Corbet et al., 2018). In addition, AC-73 disrupts CD147-MCT1 interaction, influencing MCT1 membrane localization and inhibiting lactate transport. Studies have established that AC-73 considerably suppresses tumor growth and metastasis in hepatocellular carcinoma models and augments the sensitivity to cytarabine or arsenic trioxide in leukemia (Fu et al., 2016; Spinello et al., 2019).

Nonetheless, nonspecific inhibition of lactate metabolism may cause side effects. For instance, suppressing its production may result in pyruvate accumulation, inducing collagen hydroxylation and extracellular matrix remodeling, facilitating metastasis in breast cancer (Elia et al., 2019).

Targeting the enzymes involved in lactylation modification is yet another potential antitumor strategy. The KAT8-specific inhibitor MG149 weakens eEF1A2 lactylation, inhibits CRC proliferation and metastasis, and exerts synergistic effects when combined with 5-fluorouracil chemotherapy (Xie et al., 2024). β-alanine competes with lactate for AARS1 binding, as a result of which it inhibits p53 lactylation and augments doxorubicin efficacy (Zong et al., 2024). Another promising strategy is to activate lactylation “erasers” to promote delactylation. Honokiol, a SIRT3 agonist, reduces cyclin E2 lactylation levels and effectively inhibits tumor proliferation (Jin et al., 2023). However, maintaining high lactylation levels of certain proteins may be beneficial in specific cases. Copper ions stimulate the binding of AARS1/AARS2 to METTL16, enhancing its lactylation and inducing cuproptosis. The co-administration of the copper carrier elesclomol and the SIRT2 inhibitor AGK2 increases the efficacy of elesclomol (Sun L. et al., 2023). As copper is primarily absorbed in the stomach and the upper digestive tract, delactylation enzyme inhibitors may be used as adjuvants in treating gastrointestinal tumors and mucoid adenomas (with high copper concentrations).

Beyond modulating the levels of lactate and lactylation-modifying enzymes, targeting key lactylation sites may be efficacious. K673-peptide-3 specifically blocks MRE11 K673 lactylation, reversing CRC resistance to cisplatin and oxaliplatin (Chen Y. et al., 2024). Han et al. identified TubA (HY-N2155), a small-molecule compound targeting ABCF1-K430 lactylation. The compound inhibits the binding of ABCF1-K430 to the KDM3A promoter and suppresses hepatocellular carcinoma organoid growth (Hong et al., 2025). Chen et al. developed APOC2-K70-la antibody and demonstrated that it considerably reduced tumor size, increased tumor-infiltrating CD8^+^ T cells, and markedly enhanced immunotherapeutic efficacy when co-administered with anti-PD-1 mAb (Chen J. et al., 2024).

Despite encouraging preclinical findings (Yang et al., 2021; Halford et al., 2023; Kumagai et al., 2022; Beloueche-Babari et al., 2017; García-Cañaveras et al., 2019; Apost et al., 2022; Li et al., 2022), several challenges hinder the clinical translation of lactylation-targeted therapies. First, specificity remains a major concern, as key enzymes like p300/CBP and AARS1 have multifunctional roles, raising the risk of off-target effects and toxicity. Second, site-specific lactylation inhibitors are still underdeveloped, and effective delivery to relevant subcellular compartments is technically challenging. Third, reliable lactylation biomarkers are needed for patient stratification to identify responders and minimize overtreatment. Finally, the reversible nature of lactylation may require sustained intervention, potentially increasing toxicity.

Further research is needed to determine whether the specific mechanism by which lactate affects tumors and the tumor microenvironment involves nonhistone lactylation modification. Furthermore, the specific conditions and mechanisms by which multifunctional enzymes catalyze lactylation should be investigated. Elucidating the spectrum of lactylation target proteins and their dynamic changes in various tumor microenvironments will provide a foundation to comprehend the precise role of lactate and lactylation in tumor progression. Developing highly selective and low-toxicity interventions targeting key nonhistone lactylation sites may be beneficial in overcoming the therapeutic resistance of tumors.

## 8 Conclusion

Lactylation is acknowledged as a critical PTM of histones; however, nonhistone proteins surpass histones in terms of diversity and abundance, performing more specialized functions in cellular processes. This review elucidates the lactylation mechanisms of nonhistones and their functional impacts, summarizing the significance of nonhistone lactylation in tumor metabolism, immune evasion, DNA repair, and therapeutic resistance.

Despite the substantial progress in understanding nonhistone lactylation, several challenges and knowledge gaps remain. For instance, although several lactyl transferases and lactyl-CoA synthetases have been identified, the substrate specificity mechanisms of these multifunctional enzymes remain unclear. Additional investigations are required to determine the conditions under which specific enzymes recognize particular substrates at distinct subcellular locations to catalyze lactylation rather than other processes.

In addition, most studies have focused on individual lactylation sites on specific proteins, potentially overlooking the complex interplay between multiple lactylation sites within the same protein or between different lactylated proteins within functional networks. Moreover, lactylation interacts with other PTMs. Enhanced acetylation and PDHA1 inactivation stimulate lactate accumulation, increasing the lactylation of mitochondrial fission protein 1 (An et al., 2023). In macrophages, HMGB1 lactylation promotes its acetylation, whereas histone H3K18 lactylation competitively inhibits acetylation (Rho et al., 2023). APOC2 K70 lactylation competitively inhibits ubiquitination at this site (Chen J. et al., 2024). Mounting evidence suggests that the activation of complex multiple biological effects likely creates competitive relationships among these lysine acylations, substantially increasing the potential complexity of combinatorial modifications. The crosstalk between different types of acyl modifications remains incompletely understood, and further in-depth exploration is still needed to reveal the functional interrelationships between acylation and other PTMs. Therefore, integrating lactylomics with proteomics, metabolomics, and epigenomics in the future can aid in unraveling the crosstalk of PTMs and the metabolic-epigenetic axis. In addition, utilizing lactylation-based biomarkers to identify patients suitable for lactylation-targeted antitumor therapies may enhance the efficacy of anticancer treatment.

In conclusion, nonhistone lactylation is a key regulatory mechanism in cancer biology with substantial therapeutic implications. Despite the challenges, continued studies on this emerging field can yield novel insights into tumor pathophysiology and offer innovative strategies for cancer treatment.

## Glossary

ABCF1: ATP-Binding Cassette Transporter F1
ACSS2: Acyl-CoA Synthetase Short-Chain Family Member 2
ALDH1A3: Aldehyde Dehydrogenase 1 Family Member A3
ALDOA: Aldolase A
APOC2: Apolipoprotein C2
AARS: Alanine-tRNA Synthetase
CEACAM6: Carcinoembryonic Antigen-Related Cell Adhesion Molecule 6
CPT2: Carnitine Palmitoyltransferase 2
CRC: Colorectal Cancer
DCBLD1: Discoidin, CUB And LCCL Domain-Containing 1
eEF1A2: Eukaryotic Translation Elongation Factor 1 Alpha 2
FBXO33: F-Box Protein 33
FOXP3: Forkhead Box P3
GCLC: Glutamate-Cysteine Ligase Catalytic Subunit
GLO: Glyoxalase
GSCs: Glioblastoma Stem Cells
GSH: Glutathione
GTPSCS: GTP-Specific Succinate-CoA Synthetase
HDAC: Histone Deacetylase
HIF-1α: Hypoxia-Inducible Factor-1 Alpha
HMGB1: High-Mobility Group Box 1
IFN-β: Interferon Beta
IGF2BP3: Insulin-Like Growth Factor 2 mRNA-Binding Protein 3
Kce: N-ε(Carboxyethyl)-Lysine
KD-la: D-Lactyl-Lysine
KDM3A: Lysine Demethylase 3A
Kla: Lysine Lactylation
KL-la: L-Lactyl-Lysine
LDH: Lactate Dehydrogenase
LDHA: Lactate Dehydrogenase A
LGSH: Lactoylglutathione
m5C: 5-Methylcytosine
m6A: N6-Methyladenosine
MCT: Monocarboxylate Transporter
METTL: Methyltransferase-Like Protein
MGO: Methylglyoxal
MOESIN: Membrane-Organizing Extension Spike Protein
MRE11: Meiotic Recombination 11
NBS1: Nijmegen Breakage Syndrome 1
NMNAT1: Nicotinamide Mononucleotide Adenylyltransferase 1
NSUN2: NOP2/Sun RNA Methyltransferase 2
NUSAP1: Nucleolar And Spindle Associated Protein 1
OXSM: 3-Oxoacyl-ACP Synthase
PCK2: Phosphoenolpyruvate Carboxykinase 2
PDHA1: Pyruvate Dehydrogenase E1 Alpha 1
PD-L1: Programmed Death-Ligand 1
PFKP: Phosphofructokinase P
PTBP1: Polypyrimidine Tract-Binding Protein 1
PTM: Post-Translational Modification
SIRT: Sirtuin
TEAD: Transcriptional Enhanced Associate Domain
TFEB: Transcription Factor EB
TGF-β: Transforming Growth Factor Beta
TRIM: Tripartite Motif
WWP2: WW Domain-Containing E3 Ubiquitin Protein Ligase 2
XRCC1: X-Ray Repair Cross-Complementing Protein 1
YAP: Yes-Associated Protein
YY1: Yin Yang 1

## References

[B1] An S. Yao Y. Hu H. Wu J. Li J. Li L. (2023). PDHA1 hyperacetylation-mediated lactate overproduction promotes sepsis-induced acute kidney injury *via* Fis1 lactylation. Cell Death and Dis. 14 (7), 457. 10.1038/s41419-023-05952-4 37479690 PMC10362039

[B2] Apicella M. Giannoni E. Fiore S. Ferrari K. J. Fernández-Pérez D. Isella C. (2018). Increased lactate secretion by cancer cells sustains non-cell-autonomous adaptive resistance to MET and EGFR targeted therapies. Cell Metab. 28 (6), 848–865. 10.1016/j.cmet.2018.08.006 30174307

[B3] Apostolova P. Pearce E. L. (2022). Lactic acid and lactate: revisiting the physiological roles in the tumor microenvironment. Trends Immunol. 43 (12), 969–977. 10.1016/j.it.2022.10.005 36319537 PMC10905416

[B4] Barnes E. M. E. Xu Y. Benito A. Herendi L. Siskos A. P. Aboagye E. O. (2020). Lactic acidosis induces resistance to the pan-Akt inhibitor uprosertib in Colon cancer cells. Br. J. Cancer 122 (9), 1298–1308. 10.1038/s41416-020-0777-y 32152504 PMC7188671

[B5] Beloueche-Babari M. Wantuch S. Casals Galobart T. Koniordou M. Parkes H. G. Arunan V. (2017). MCT1 inhibitor AZD3965 increases mitochondrial metabolism, facilitating combination therapy and noninvasive magnetic resonance spectroscopy. Cancer Res. 77 (21), 5913–5924. 10.1158/0008-5472.CAN-16-2686 28923861 PMC5669455

[B6] Beloueche-Babari M. Casals Galobart T. Delgado-Goni T. Wantuch S. Parkes H. G. Tandy D. (2020). Monocarboxylate transporter 1 blockade with AZD3965 inhibits lipid biosynthesis and increases tumour immune cell infiltration. Br. J. Cancer 122 (6), 895–903. 10.1038/s41416-019-0717-x 31937921 PMC7078321

[B7] Bianchetti D. G. A. M. Amelio G. S. Lava S. A. G. Bianchetti M. G. Simonetti G. D. Agostoni C. (2018). D-lactic acidosis in humans: systematic literature review. Pediatr. Nephrol. Berl. Ger. 33 (4), 673–681. 10.1007/s00467-017-3844-8 29218437

[B8] Brooks G. A. (2018). The science and translation of lactate shuttle theory. Cell Metab. 27 (4), 757–785. 10.1016/j.cmet.2018.03.008 29617642

[B9] Cai L. Sutter B. M. Li B. Tu B. P. (2011). Acetyl-CoA induces cell growth and proliferation by promoting the acetylation of histones at growth genes. Mol. Cell 42 (4), 426–437. 10.1016/j.molcel.2011.05.004 21596309 PMC3109073

[B10] Certo M. Tsai C. H. Pucino V. Ho P. C. Mauro C. (2021). Lactate modulation of immune responses in inflammatory *versus* tumour microenvironments. Nat. Rev. Immunol. 21 (3), 151–161. 10.1038/s41577-020-0406-2 32839570

[B11] Chen M. Cen K. Song Y. Zhang X. Liou Y. C. Liu P. (2023). NUSAP1-LDHA-Glycolysis-Lactate feedforward loop promotes Warburg effect and metastasis in pancreatic ductal adenocarcinoma. Cancer Lett. 567, 216285. 10.1016/j.canlet.2023.216285 37354982

[B12] Chen Y. Wu J. Zhai L. Zhang T. Yin H. Gao H. (2024a). Metabolic regulation of homologous recombination repair by MRE11 lactylation. Cell 187 (2), 294–311.e21. 10.1016/j.cell.2023.11.022 38128537 PMC11725302

[B13] Chen H. Li Y. Li H. Chen X. Fu H. Mao D. (2024b). NBS1 lactylation is required for efficient DNA repair and chemotherapy resistance. Nature 631 (8021), 663–669. 10.1038/s41586-024-07620-9 38961290 PMC11254748

[B14] Chen J. Zhao D. Wang Y. Liu M. Zhang Y. Feng T. (2024c). Lactylated apolipoprotein C-II induces immunotherapy resistance by promoting extracellular lipolysis. Adv. Sci. Weinheim, Baden-Wurttemberg, Ger. 11 (38), e2406333. 10.1002/advs.202406333 38981044 PMC11481198

[B15] Chen R. Zou J. Zhong X. Li J. Kang R. Tang D. (2024d). HMGB1 in the interplay between autophagy and apoptosis in cancer. Cancer Lett. 581, 216494. 10.1016/j.canlet.2023.216494 38007142

[B16] Chen J. Huang Z. Chen Y. Tian H. Chai P. Shen Y. (2025a). Lactate and lactylation in cancer. Signal Transduct. Target. Ther. 10 (1), 38. 10.1038/s41392-024-02082-x 39934144 PMC11814237

[B17] Chen J. He J. Wang X. Bai L. Yang X. (2025b). Glis1 inhibits RTEC cellular senescence and renal fibrosis by downregulating histone lactylation in DKD. Life Sci. 361, 123293. 10.1016/j.lfs.2024.123293 39643036

[B18] Cheng Z. Huang H. Li M. Liang X. Tan Y. Chen Y. (2023). Lactylation-Related gene signature effectively predicts prognosis and treatment responsiveness in hepatocellular carcinoma. Pharm. Basel, Switz. 16 (5), 644. 10.3390/ph16050644 37242427 PMC10221268

[B19] Chu Y.-D. Cheng L. C. Lim S. N. Lai M. W. Yeh C. T. Lin W. R. (2023). Aldolase B-driven lactagenesis and CEACAM6 activation promote cell renewal and chemoresistance in colorectal cancer through the Warburg effect. Cell Death and Dis. 14 (10), 660. 10.1038/s41419-023-06187-z 37816733 PMC10564793

[B20] Corbet C. Bastien E. Draoui N. Doix B. Mignion L. Jordan B. F. (2018). Interruption of lactate uptake by inhibiting mitochondrial pyruvate transport unravels direct antitumor and radiosensitizing effects. Nat. Commun. 9 (1), 1208. 10.1038/s41467-018-03525-0 29572438 PMC5865202

[B21] Dai E. Wang W. Li Y. Ye D. Li Y. (2024). Lactate and lactylation: behind the development of tumors. Cancer Lett. 591, 216896. 10.1016/j.canlet.2024.216896 38641309

[B22] Doherty J. R. Cleveland J. L. (2013). Targeting lactate metabolism for cancer therapeutics. J. Clin. Investigation 123 (9), 3685–3692. 10.1172/JCI69741 23999443 PMC3754272

[B23] Draoui N. Schicke O. Seront E. Bouzin C. Sonveaux P. Riant O. (2014). Antitumor activity of 7-aminocarboxycoumarin derivatives, a new class of potent inhibitors of lactate influx but not efflux. Mol. Cancer Ther. 13 (6), 1410–1418. 10.1158/1535-7163.MCT-13-0653 24672058

[B24] Duan Y. Zhan H. Wang Q. Li B. Gao H. Liu D. (2024). Integrated lactylome characterization reveals the molecular dynamics of protein regulation in gastrointestinal cancers. Adv. Sci. Weinheim, Baden-Wurttemberg, Ger. 11 (35), e2400227. 10.1002/advs.202400227 39018247 PMC11425215

[B25] Duxbury M. S. Ito H. Benoit E. Waseem T. Ashley S. W. Whang E. E. (2004). A novel role for carcinoembryonic antigen-related cell adhesion molecule 6 as a determinant of gemcitabine chemoresistance in pancreatic adenocarcinoma cells. Cancer Res. 64 (11), 3987–3993. 10.1158/0008-5472.CAN-04-0424 15173012

[B26] Elia I. Rossi M. Stegen S. Broekaert D. Doglioni G. van Gorsel M. (2019). Breast cancer cells rely on environmental pyruvate to shape the metastatic niche. Nature 568 (7750), 117–121. 10.1038/s41586-019-0977-x 30814728 PMC6451642

[B27] Fan H. Yang F. Xiao Z. Luo H. Chen H. Chen Z. (2023a). Lactylation: novel epigenetic regulatory and therapeutic opportunities. Am. J. Physiology. Endocrinol. Metabolism 324 (4), E330–E338. 10.1152/ajpendo.00159.2022 36856188

[B28] Fan M. Yang K. Wang X. Chen L. Gill P. S. Ha T. (2023b). Lactate promotes endothelial-to-mesenchymal transition *via* Snail1 lactylation after myocardial infarction. Sci. Adv. 9 (5), eadc9465. 10.1126/sciadv.adc9465 36735787 PMC9897666

[B29] Fan W. Wang X. Zeng S. Wang G. Li R. (2023c). Global lactylome reveals lactylation-dependent mechanisms underlying TH17 differentiation in experimental autoimmune uveitis. Sci. Adv. 9 (42), eadh4655. 10.1126/sciadv.adh4655 37851814 PMC10584346

[B30] Feng F. Wu J. Chi Q. Wang S. Liu W. Yang L. (2024). Lactylome analysis unveils Lactylation-Dependent mechanisms of stemness remodeling in the liver cancer stem cells. Adv. Sci. Weinheim, Baden-Wurttemberg, Ger. 11 (38), e2405975. 10.1002/advs.202405975 39099416 PMC11481176

[B31] Fischer K. Hoffmann P. Voelkl S. Meidenbauer N. Ammer J. Edinger M. (2007). Inhibitory effect of tumor cell-derived lactic acid on human T cells. Blood 109 (9), 3812–3819. 10.1182/blood-2006-07-035972 17255361

[B32] Fu Z.-g. Wang L. Cui H. y. Peng J. l. Wang S. j. Geng J. j. (2016). A novel small-molecule compound targeting CD147 inhibits the motility and invasion of hepatocellular carcinoma cells. Oncotarget 7 (8), 9429–9447. 10.18632/oncotarget.6990 26882566 PMC4891050

[B33] Gaffney D. O. Jennings E. Q. Anderson C. C. Marentette J. O. Shi T. Schou Oxvig A. M. (2020). Non-enzymatic lysine lactoylation of glycolytic enzymes. Cell Chem. Biol. 27 (2), 206–213. 10.1016/j.chembiol.2019.11.005 31767537 PMC7395678

[B34] Galligan J. J. Wepy J. A. Streeter M. D. Kingsley P. J. Mitchener M. M. Wauchope O. R. (2018). Methylglyoxal-derived posttranslational arginine modifications are abundant histone marks. Proc. Natl. Acad. Sci. U. S. A. 115 (37), 9228–9233. 10.1073/pnas.1802901115 30150385 PMC6140490

[B35] Ganapathy-Kanniappan S. Geschwind J-. F. H. (2013). “Tumor glycolysis as a target for cancer therapy: progress and prospects. Mol. Cancer 12, 152. 10.1186/1476-4598-12-152 24298908 PMC4223729

[B36] Gao Q. Zhu H. Dong L. Shi W. Chen R. Song Z. (2019). Integrated proteogenomic characterization of HBV-Related hepatocellular carcinoma. Cell 179 (5), 1240. 10.1016/j.cell.2019.10.038 31730861

[B37] Gao X. Pang C. Fan Z. Wang Y. Duan Y. Zhan H. (2024). Regulation of newly identified lysine lactylation in cancer. Cancer Lett. 587, 216680. 10.1016/j.canlet.2024.216680 38346584

[B38] García-Cañaveras J. C. Chen L. Rabinowitz J. D. (2019). The Tumor metabolic microenvironment: lessons from lactate. Cancer Res. 79 (13), 3155–3162. 10.1158/0008-5472.CAN-18-3726 31171526 PMC6606343

[B39] Gu J. Zhou J. Chen Q. Xu X. Gao J. Li X. (2022). Tumor metabolite lactate promotes tumorigenesis by modulating MOESIN lactylation and enhancing TGF-β signaling in regulatory T cells. Cell Rep. 39 (12), 110986. 10.1016/j.celrep.2022.110986 35732125

[B40] Guan X. Morris M. E. (2020). *In vitro* and *in vivo* efficacy of AZD3965 and Alpha-Cyano-4-Hydroxycinnamic acid in the Murine 4T1 breast tumor model. AAPS J. 22 (4), 84. 10.1208/s12248-020-00466-9 32529599 PMC8066402

[B41] Halford S. Veal G. J. Wedge S. R. Payne G. S. Bacon C. M. Sloan P. (2023). A phase I dose-escalation Study of AZD3965, an oral monocarboxylate transporter 1 inhibitor, in patients with advanced cancer. Clin. Cancer Res. Official J. Am. Assoc. For Cancer Res. 29 (8), 1429–1439. 10.1158/1078-0432.CCR-22-2263 36652553 PMC7614436

[B42] Hanahan D. Weinberg R. A. (2011). Hallmarks of cancer: the next generation. Cell 144 (5), 646–674. 10.1016/j.cell.2011.02.013 21376230

[B43] He Y. Song T. Ning J. Wang Z. Yin Z. Jiang P. (2024). Lactylation in cancer: mechanisms in tumour biology and therapeutic potentials. Clin. Transl. Med. 14 (11), e70070. 10.1002/ctm2.70070 39456119 PMC11511673

[B44] Hong H. Han H. Wang L. Cao W. Hu M. Li J. (2025). ABCF1-K430-Lactylation promotes HCC malignant progression *via* transcriptional activation of HIF1 signaling pathway. Cell Death Differ. 32 (4), 613–631. 10.1038/s41418-024-01436-w 39753865 PMC11982231

[B45] Hu X. Huang X. Yang Y. Sun Y. Zhao Y. Zhang Z. (2024). Dux activates metabolism-lactylation-MET network during early iPSC reprogramming with Brg1 as the histone lactylation reader. Nucleic Acids Res. 52 (10), 5529–5548. 10.1093/nar/gkae183 38512058 PMC11162783

[B46] Huang Y. Luo G. Peng K. Song Y. Wang Y. Zhang H. (2024a). Lactylation stabilizes TFEB to elevate autophagy and lysosomal activity. J. Cell Biol. 223 (11), e202308099. 10.1083/jcb.202308099 39196068 PMC11354204

[B47] Huang H. Wang S. Xia H. Zhao X. Chen K. Jin G. (2024b). Lactate enhances NMNAT1 lactylation to sustain nuclear NAD+ salvage pathway and promote survival of pancreatic adenocarcinoma cells under glucose-deprived conditions. Cancer Lett. 588, 216806. 10.1016/j.canlet.2024.216806 38467179

[B48] Jia M. Yue X. Sun W. Zhou Q. Chang C. Gong W. (2023). ULK1-mediated metabolic reprogramming regulates Vps34 lipid kinase activity by its lactylation. Sci. Adv. 9 (22), eadg4993. 10.1126/sciadv.adg4993 37267363 PMC10413652

[B49] Jin J. Bai L. Wang D. Ding W. Cao Z. Yan P. (2023). SIRT3-dependent delactylation of cyclin E2 prevents hepatocellular carcinoma growth. EMBO Rep. 24 (5), e56052. 10.15252/embr.202256052 36896611 PMC10157311

[B50] Ju J. Zhang H. Lin M. Yan Z. Cao Z. (2024). The alanyl-tRNA synthetase AARS1 moonlights as a lactyltransferase to promote YAP signaling in gastric cancer. J. Clin. Investigation 134 (10), e174587. 10.1172/JCI174587 38512451 PMC11093599

[B51] Kim Y. H. Kwak M. S. Lee B. Shin J. M. Aum S. Park I. H. (2021). Secretory autophagy machinery and vesicular trafficking are involved in HMGB1 secretion. Autophagy 17 (9), 2345–2362. 10.1080/15548627.2020.1826690 33017561 PMC8496717

[B52] Kumagai S. Togashi Y. Sakai C. Kawazoe A. Kawazu M. Ueno T. (2020). An oncogenic alteration creates a microenvironment that promotes tumor progression by conferring a metabolic advantage to regulatory T cells. Immunity 53 (1), 187–203. 10.1016/j.immuni.2020.06.016 32640259

[B53] Kumagai S. Koyama S. Itahashi K. Tanegashima T. Lin Y. T. Togashi Y. (2022). Lactic acid promotes PD-1 expression in regulatory T cells in highly glycolytic tumor microenvironments. Cancer Cell 40 (2), 201–218.e9. 10.1016/j.ccell.2022.01.001 35090594

[B54] Le A. Cooper C. R. Gouw A. M. Dinavahi R. Maitra A. Deck L. M. (2010). Inhibition of lactate dehydrogenase A induces oxidative stress and inhibits tumor progression. Proc. Natl. Acad. Sci. U. S. A. 107 (5), 2037–2042. 10.1073/pnas.0914433107 20133848 PMC2836706

[B55] Levitt M. D. Levitt D. G. (2020). Quantitative evaluation of D-Lactate pathophysiology: new insights into the mechanisms involved and the many areas in need of further investigation. Clin. Exp. Gastroenterology 13, 321–337. 10.2147/CEG.S260600 32982363 PMC7490090

[B56] Li L. Chen K. Wang T. Wu Y. Xing G. Chen M. (2020). Glis1 facilitates induction of pluripotency *via* an epigenome-metabolome-epigenome signalling cascade. Nat. Metab. 2 (9), 882–892. 10.1038/s42255-020-0267-9 32839595

[B57] Li X. Yang Y. Zhang B. Lin X. Fu X. An Y. (2022). Lactate metabolism in human health and disease. Signal Transduct. Target. Ther. 7 (1), 305. 10.1038/s41392-022-01151-3 36050306 PMC9434547

[B58] Li H. Liu C. Li R. Zhou L. Yang Q. (2024a). AARS1 and AARS2 sense L-lactate to regulate cGAS as global lysine lactyltransferases. Nature 634 (8036), 1229–1237. 10.1038/s41586-024-07992-y 39322678

[B59] Li G. Wang D. Zhai Y. Pan C. Zhang J. Wang C. (2024b). Glycometabolic reprogramming-induced XRCC1 lactylation confers therapeutic resistance in ALDH1A3-overexpressing glioblastoma. Cell Metab. 36 (8), 1696–1710.e10. 10.1016/j.cmet.2024.07.011 39111285

[B60] Li Y. Min X. Zhang X. Cao X. Kong Q. Mao Q. (2024c). HSPA12A promotes c-Myc lactylation-mediated proliferation of tubular epithelial cells to facilitate renal functional recovery from kidney ischemia/reperfusion injury. Cell. Mol. Life Sci. CMLS 81 (1), 404. 10.1007/s00018-024-05427-5 39277835 PMC11402889

[B61] Li T. Xu D. Ruan Z. Zhou J. Sun W. Rao B. (2024d). Metabolism/Immunity dual-regulation thermogels potentiating immunotherapy of glioblastoma through lactate-excretion inhibition and PD-1/PD-L1 blockade. Adv. Sci. Weinheim, Baden-Wurttemberg, Ger. 11 (18), e2310163. 10.1002/advs.202310163 38460167 PMC11095231

[B62] Liu R. Wu J. Guo H. Yao W. Li S. Lu Y. (2023). Post-translational modifications of histones: mechanisms, biological functions, and therapeutic targets. MedComm 4 (3), e292. 10.1002/mco2.292 37220590 PMC10200003

[B63] Liu R. Ren X. Park Y. E. Feng H. Sheng X. Song X. (2025). Nuclear GTPSCS functions as a lactyl-CoA synthetase to promote histone lactylation and gliomagenesis. Cell Metab. 37 (2), 377–394.e9. 10.1016/j.cmet.2024.11.005 39642882 PMC11798710

[B64] Locati M. Curtale G. Mantovani A. (2020). Diversity, mechanisms, and significance of macrophage plasticity. Annu. Rev. Pathology 15, 123–147. 10.1146/annurev-pathmechdis-012418-012718 31530089 PMC7176483

[B65] Lopez E. Karattil R. Nannini F. Weng-Kit Cheung G. Denzler L. Galvez-Cancino F. (2023). Inhibition of lactate transport by MCT-1 blockade improves chimeric antigen receptor T-cell therapy against B-cell malignancies. J. For Immunother. Cancer 11 (6), e006287. 10.1136/jitc-2022-006287 37399358 PMC10314680

[B66] Lu Y. Zhu J. Zhang Y. Li W. Xiong Y. Fan Y. (2024). Lactylation-Driven IGF2BP3-Mediated serine metabolism reprogramming and RNA m6A-Modification promotes lenvatinib resistance in HCC. Adv. Sci. Weinheim, Baden-Wurttemberg, Ger. 11 (46), e2401399. 10.1002/advs.202401399 39450426 PMC11633555

[B67] Luo Y. Yang Z. Yu Y. Zhang P. (2022). HIF1α lactylation enhances KIAA1199 transcription to promote angiogenesis and vasculogenic mimicry in prostate cancer. Int. J. Biol. Macromol. 222 (Pt B), 2225–2243. 10.1016/j.ijbiomac.2022.10.014 36209908

[B68] Lv X. Lv Y. Dai X. (2023). Lactate, histone lactylation and cancer hallmarks. Expert Rev. Mol. Med. 25, e7. 10.1017/erm.2022.42 36621008

[B69] Mao Y. Zhang J. Zhou Q. He X. Zheng Z. Wei Y. (2024). Hypoxia induces mitochondrial protein lactylation to limit oxidative phosphorylation. Cell Res. 34 (1), 13–30. 10.1038/s41422-023-00864-6 38163844 PMC10770133

[B70] Meng Q. Sun H. Zhang Y. Yang X. Hao S. Liu B. (2024). Lactylation stabilizes DCBLD1 activating the pentose phosphate pathway to promote cervical cancer progression. J. Exp. and Clin. Cancer Res. CR 43 (1), 36. 10.1186/s13046-024-02943-x 38291438 PMC10829273

[B71] Mi J. Zhao L. Shen Y. Mo S. Kuang Y. (2024). PFKP lactylation promotes the ovarian cancer progression through targeting PTEN. Biochem. Genet. 10.1007/s10528-024-10990-4 39638933

[B72] Miao Z. Zhao X. Liu X. (2023). Hypoxia induced β-catenin lactylation promotes the cell proliferation and stemness of colorectal cancer through the wnt signaling pathway. Exp. Cell Res. 422 (1), 113439. 10.1016/j.yexcr.2022.113439 36464122

[B73] Moreno-Yruela C. Zhang D. Wei W. Bæk M. Liu W. Gao J. (2022). Class I histone deacetylases (HDAC1-3) are histone lysine delactylases. Sci. Adv. 8 (3), eabi6696. 10.1126/sciadv.abi6696 35044827 PMC8769552

[B74] Niu Z. Chen C. Wang S. Lu C. Wu Z. Wang A. (2024). HBO1 catalyzes lysine lactylation and mediates histone H3K9la to regulate gene transcription. Nat. Commun. 15 (1), 3561. 10.1038/s41467-024-47900-6 38670996 PMC11053077

[B75] Niu K. Chen Z. Li M. Ma G. Deng Y. Zhang J. (2025). NSUN2 lactylation drives cancer cell resistance to ferroptosis through enhancing GCLC-dependent glutathione synthesis. Redox Biol. 79, 103479. 10.1016/j.redox.2024.103479 39742570 PMC11750563

[B76] Noble R. A. Bell N. Blair H. Sikka A. Thomas H. Phillips N. (2017). Inhibition of monocarboxyate transporter 1 by AZD3965 as a novel therapeutic approach for diffuse large B-cell lymphoma and Burkitt lymphoma. Haematologica 102 (7), 1247–1257. 10.3324/haematol.2016.163030 28385782 PMC5566036

[B77] Nuñez R. Sidlowski P. F. W. Steen E. A. Wynia-Smith S. L. Sprague D. J. Keyes R. F. (2024). The TRIM33 bromodomain recognizes histone lysine lactylation. ACS Chem. Biol. 19 (12), 2418–2428. 10.1021/acschembio.4c00248 39556662 PMC11706526

[B78] Pan R.-Y. Zhang J. Liu X. Liao Y. Gao J. (2022). Positive feedback regulation of microglial glucose metabolism by histone H4 lysine 12 lactylation in Alzheimer's disease. Cell Metab. 34 (4), 634–648.e6. 10.1016/j.cmet.2022.02.013 35303422

[B79] Pouysségur J. Marchiq I. Parks S. K. Durivault J. Ždralević M. Vucetic M. (2022). Warburg effect' controls tumor growth, bacterial, viral infections and immunity - genetic deconstruction and therapeutic perspectives. Seminars Cancer Biol. 86 (Pt 2), 334–346. 10.1016/j.semcancer.2022.07.004 35820598

[B80] Rho H. Terry A. R. Chronis C. Hay N. (2023). Hexokinase 2-mediated gene expression *via* histone lactylation is required for hepatic stellate cell activation and liver fibrosis. Cell Metab. 35 (8), 1406–1423.e8. 10.1016/j.cmet.2023.06.013 37463576 PMC11748916

[B81] Rizeq B. Zakaria Z. Ouhtit A. (2018). Towards understanding the mechanisms of actions of carcinoembryonic antigen-related cell adhesion molecule 6 in cancer progression. Cancer Sci. 109 (1), 33–42. 10.1111/cas.13437 29110374 PMC5765285

[B82] Sabari B. R. Zhang D. Allis C. D. Zhao Y. (2017). Metabolic regulation of gene expression through histone acylations. Nat. Rev. Mol. Cell Biol. 18 (2), 90–101. 10.1038/nrm.2016.140 27924077 PMC5320945

[B83] Shao C. Tang S. Yu S. Liu C. Zhang Y. Wan T. (2025). Genetic code expansion reveals site-specific lactylation in living cells reshapes protein functions. Nat. Commun. 16 (1), 227. 10.1038/s41467-024-55165-2 39779673 PMC11711764

[B84] Sharma D. Singh M. Rani R. (2022). Role of LDH in tumor glycolysis: regulation of LDHA by small molecules for cancer therapeutics. Seminars Cancer Biol. 87, 184–195. 10.1016/j.semcancer.2022.11.007 36371026

[B85] Shinde-Jadhav S. Mansure J. J. Rayes R. F. Marcq G. Ayoub M. Skowronski R. (2021). Role of neutrophil extracellular traps in radiation resistance of invasive bladder cancer. Nat. Commun. 12 (1), 2776. 10.1038/s41467-021-23086-z 33986291 PMC8119713

[B86] Spinello I. Saulle E. Quaranta M. T. Pasquini L. Pelosi E. Castelli G. (2019). The small-molecule compound AC-73 targeting CD147 inhibits leukemic cell proliferation, induces autophagy and increases the chemotherapeutic sensitivity of acute myeloid leukemia cells. Haematologica 104 (5), 973–985. 10.3324/haematol.2018.199661 30467201 PMC6518905

[B87] Stacpoole P. W. Dirain C. O. (2024). The pyruvate dehydrogenase complex at the epigenetic crossroads of acetylation and lactylation. Mol. Genet. Metabolism 143 (1-2), 108540. 10.1016/j.ymgme.2024.108540 39067348

[B88] Sun Y. Chen Y. Peng T. (2022). A bioorthogonal chemical reporter for the detection and identification of protein lactylation. Chem. Sci. 13 (20), 6019–6027. 10.1039/d2sc00918h 35685793 PMC9132054

[B89] Sun W. Jia M. Feng Y. Cheng X. (2023a). Lactate is a bridge linking glycolysis and autophagy through lactylation. Autophagy 19 (12), 3240–3241. 10.1080/15548627.2023.2246356 37565742 PMC10621282

[B90] Sun L. Zhang Y. Yang B. Sun S. Zhang P. Luo Z. (2023b). Lactylation of METTL16 promotes cuproptosis *via* m6A-modification on FDX1 mRNA in gastric cancer. Nat. Commun. 14 (1), 6523. 10.1038/s41467-023-42025-8 37863889 PMC10589265

[B91] Tong H. Jiang Z. Song L. Tan K. Yin X. He C. (2024). Dual impacts of serine/glycine-free diet in enhancing antitumor immunity and promoting evasion *via* PD-L1 lactylation. Cell Metab. 36 (12), 2493–2510.e9. 10.1016/j.cmet.2024.10.019 39577415

[B92] Urbano A. M. (2021). Otto warburg: the journey towards the seminal discovery of tumor cell bioenergetic reprogramming. Biochimica Biophysica Acta. Mol. Basis Dis. 1867 (1), 165965. 10.1016/j.bbadis.2020.165965 32949769

[B93] Vander Heiden M. G. Cantley L. C. Thompson C. B. (2009). Understanding the warburg effect: the metabolic requirements of cell proliferation. Sci. (New York, N.Y.) 324 (5930), 1029–1033. 10.1126/science.1160809 19460998 PMC2849637

[B94] Wan N. Wang N. Yu S. Zhang H. Tang S. Wang D. (2022). Cyclic immonium ion of lactyllysine reveals widespread lactylation in the human proteome. Nat. Methods 19 (7), 854–864. 10.1038/s41592-022-01523-1 35761067

[B95] Wang N. Wang W. Wang X. Mang G. Chen J. Yan X. (2022a). Histone lactylation boosts reparative gene activation post-myocardial infarction. Circulation Res. 131 (11), 893–908. 10.1161/CIRCRESAHA.122.320488 36268709

[B96] Wang J. Yang P. Yu T. Gao M. Liu D. Zhang J. (2022b). Lactylation of PKM2 suppresses inflammatory metabolic adaptation in pro-inflammatory macrophages. Int. J. Biol. Sci. 18 (16), 6210–6225. 10.7150/ijbs.75434 36439872 PMC9682528

[B97] Wang X. Fan W. Ma Y. Yao M. Wang G. (2023a). YY1 lactylation in microglia promotes angiogenesis through transcription activation-mediated upregulation of FGF2. Genome Biol. 24 (1), 87. 10.1186/s13059-023-02931-y 37085894 PMC10120156

[B98] Wang Z.-H. Zhang P. Peng W. B. Ye L. L. Xiang X. Wei X. S. (2023b). Altered phenotypic and metabolic characteristics of FOXP3+CD3+CD56+ natural killer T (NKT)-Like cells in human malignant pleural effusion. Oncoimmunology 12 (1), 2160558. 10.1080/2162402X.2022.2160558 36567801 PMC9788685

[B99] Wang Y.-H. Gao P. Xu L. Z. Zeng K. W. Tu P. F. (2024). Small-molecule targeting PKM2 provides a molecular basis of lactylation-dependent fibroblast-like synoviocytes proliferation inhibition against rheumatoid arthritis. Eur. J. Pharmacol. 972, 176551. 10.1016/j.ejphar.2024.176551 38570082

[B100] Weng W. He Z. Ma Z. Huang J. Han Y. Feng Q. (2025). Tufm lactylation regulates neuronal apoptosis by modulating mitophagy in traumatic brain injury. Cell Death Differ. 32 (3), 530–545. 10.1038/s41418-024-01408-0 39496783 PMC11894137

[B101] Wu G. Fang Y. Z. Yang S. Lupton J. R. Turner N. D. (2004). Glutathione metabolism and its implications for health. J. Nutr. 134 (3), 489–492. 10.1093/jn/134.3.489 14988435

[B102] Wu H. Wang Y. Ying M. Jin C. Li J. Hu X. (2021). Lactate dehydrogenases amplify reactive oxygen species in cancer cells in response to oxidative stimuli. Signal Transduct. Target. Ther. 6 (1), 242. 10.1038/s41392-021-00595-3 34176927 PMC8236487

[B103] Wu Z. Peng Y. Chen W. Xia F. Song T. Ke Q. (2025). Lactylation-driven transcriptional activation of FBXO33 promotes gallbladder cancer metastasis by regulating p53 polyubiquitination. Cell Death and Dis. 16 (1), 144. 10.1038/s41419-025-07372-y 40021626 PMC11871038

[B104] Xie B. Zhang M. Li J. Cui J. Zhang P. Liu F. (2024). KAT8-catalyzed lactylation promotes eEF1A2-mediated protein synthesis and colorectal carcinogenesis. Proc. Natl. Acad. Sci. U. S. A. 121 (8), e2314128121. 10.1073/pnas.2314128121 38359291 PMC10895275

[B105] Xiong J. He J. Zhu J. Pan J. Liao W. Ye H. (2022). Lactylation-driven METTL3-mediated RNA m6A modification promotes immunosuppression of tumor-infiltrating myeloid cells. Mol. Cell 82 (9), 1660–1677.e10. 10.1016/j.molcel.2022.02.033 35320754

[B106] Xu K. Zhang K. Wang Y. Gu Y. (2024a). Comprehensive review of histone lactylation: structure, function, and therapeutic targets. Biochem. Pharmacol. 225, 116331. 10.1016/j.bcp.2024.116331 38821374

[B107] Xu Y. Ma X. Ni W. Zheng L. Lin Z. Lai Y. (2024b). PKM2-Driven lactate overproduction triggers endothelial-to-mesenchymal transition in ischemic flap *via* mediating TWIST1 lactylation. Adv. Sci. Weinheim, Baden-Wurttemberg, Ger. 11 (47), e2406184. 10.1002/advs.202406184 39474980 PMC11653614

[B108] Yan Q. Zhou J. Gu Y. Huang W. Ruan M. Zhang H. (2024). Lactylation of NAT10 promotes N4-acetylcytidine modification on tRNASer-CGA-1-1 to boost oncogenic DNA virus KSHV reactivation. Cell Death Differ. 31 (10), 1362–1374. 10.1038/s41418-024-01327-0 38879723 PMC11445560

[B109] Yang Y. Chong Y. Chen M. Dai W. Zhou X. Ji Y. (2021). Targeting lactate dehydrogenase a improves radiotherapy efficacy in non-small cell lung cancer: from bedside to bench. J. Transl. Med. 19 (1), 170. 10.1186/s12967-021-02825-2 33902615 PMC8074241

[B110] Yang K. Fan M. Wang X. Xu J. Wang Y. Tu F. (2022). Lactate promotes macrophage HMGB1 lactylation, acetylation, and exosomal release in polymicrobial sepsis. Cell Death Differ. 29 (1), 133–146. 10.1038/s41418-021-00841-9 34363018 PMC8738735

[B111] Yang H. Zou X. Yang S. Zhang A. Ma Z. (2023a). Identification of lactylation related model to predict prognostic, tumor infiltrating immunocytes and response of immunotherapy in gastric cancer. Front. Immunol. 14, 1149989. 10.3389/fimmu.2023.1149989 36936929 PMC10020516

[B112] Yang Z. Yan C. Ma J. Peng P. Ren X. Cai S. (2023b). Lactylome analysis suggests lactylation-dependent mechanisms of metabolic adaptation in hepatocellular carcinoma. Nat. Metab. 5 (1), 61–79. 10.1038/s42255-022-00710-w 36593272

[B113] Yang L. Niu K. Wang J. Shen W. Jiang R. Liu L. (2024). Nucleolin lactylation contributes to intrahepatic cholangiocarcinoma pathogenesis *via* RNA splicing regulation of MADD. J. Hepatology 81 (4), 651–666. 10.1016/j.jhep.2024.04.010 38679071

[B114] Ye L. Jiang Y. Zhang M. (2022). Crosstalk between glucose metabolism, lactate production and immune response modulation. Cytokine and Growth Factor Rev. 68, 81–92. 10.1016/j.cytogfr.2022.11.001 36376165

[B115] Yu J. Chai P. Xie M. Ge S. Ruan J. Fan X. (2021). Histone lactylation drives oncogenesis by facilitating m6A reader protein YTHDF2 expression in ocular melanoma. Genome Biol. 22 (1), 85. 10.1186/s13059-021-02308-z 33726814 PMC7962360

[B116] Yuan J. Yang M. Wu Z. Wu J. Zheng K. Wang J. (2025). The lactate-primed KAT8‒PCK2 axis exacerbates hepatic ferroptosis during ischemia/reperfusion injury by reprogramming OXSM-dependent mitochondrial fatty acid synthesis. Adv. Sci. Weinheim, Baden-Wurttemberg, Ger. 12, e2414141. 10.1002/advs.202414141 39853940 PMC11923996

[B117] Zessin M. Meleshin M. Praetorius L. Sippl W. Bařinka C. Schutkowski M. (2022). Uncovering robust delactoylase and depyruvoylase activities of HDAC isoforms. ACS Chem. Biol. 17 (6), 1364–1375. 10.1021/acschembio.1c00863 35639992

[B118] Zhai G. Niu Z. Jiang Z. Zhao F. Wang S. Chen C. (2024). DPF2 reads histone lactylation to drive transcription and tumorigenesis. Proc. Natl. Acad. Sci. U. S. A. 121 (50), e2421496121. 10.1073/pnas.2421496121 39636855 PMC11648877

[B119] Zhang D. Tang Z. Huang H. Zhou G. Cui C. Weng Y. (2019). Metabolic regulation of gene expression by histone lactylation. Nature 574 (7779), 575–580. 10.1038/s41586-019-1678-1 31645732 PMC6818755

[B120] Zhang N. Zhang Y. Xu J. Wang P. Wu B. Lu S. (2023a). α-myosin heavy chain lactylation maintains sarcomeric structure and function and alleviates the development of heart failure. Cell Res. 33 (9), 679–698. 10.1038/s41422-023-00844-w 37443257 PMC10474270

[B121] Zhang W. Xu L. Yu Z. Zhang M. Liu J. Zhou J. (2023b). Inhibition of the glycolysis prevents the cerebral infarction progression through decreasing the lactylation levels of LCP1. Mol. Biotechnol. 65 (8), 1336–1345. 10.1007/s12033-022-00643-5 36574182 PMC10352161

[B122] Zhang K. K. Burns C. M. Skinner M. E. Lombard D. B. Miller R. A. Endicott S. J. (2023c). PTEN is both an activator and a substrate of chaperone-mediated autophagy. J. Cell Biol. 222 (9), e202208150. 10.1083/jcb.202208150 37418003 PMC10327811

[B123] Zhang Y. Song H. Li M. Lu P. (2024). Histone lactylation bridges metabolic reprogramming and epigenetic rewiring in driving carcinogenesis: oncometabolite fuels oncogenic transcription. Clin. Transl. Med. 14 (3), e1614. 10.1002/ctm2.1614 38456209 PMC10921234

[B124] Zhang D. Gao J. Zhu Z. Mao Q. Xu Z. Singh P. K. (2025). Lysine L-lactylation is the dominant lactylation isomer induced by glycolysis. Nat. Chem. Biol. 21 (1), 91–99. 10.1038/s41589-024-01680-8 39030363 PMC11666458

[B125] Zhao Y. Yao X. Fei Y. Lin Z. Li Z. (2020). HCAR1/MCT1 regulates tumor ferroptosis through the lactate-mediated AMPK-SCD1 activity and its therapeutic implications. Cell Rep. 33 (10), 108487. 10.1016/j.celrep.2020.108487 33296645

[B126] Zhao Q. Wang Q. Yao Q. Yang Z. Li W. Cheng X. (2025). Nonenzymatic lysine D-lactylation induced by glyoxalase II substrate SLG dampens inflammatory immune responses. Cell Res. 35 (2), 97–116. 10.1038/s41422-024-01060-w 39757301 PMC11770101

[B127] Zhou Z. Yin X. Sun H. Lu J. Li Y. Fan Y. (2025). PTBP1 lactylation promotes glioma stem cell maintenance through PFKFB4-Driven glycolysis. Cancer Res. 85 (4), 739–757. 10.1158/0008-5472.CAN-24-1412 39570804

[B128] Zhu R. Ye X. Lu X. Xiao L. Yuan M. Zhao H. (2025). ACSS2 acts as a lactyl-CoA synthetase and couples KAT2A to function as a lactyltransferase for histone lactylation and tumor immune evasion. Cell Metab. 37 (2), 361–376.e7. 10.1016/j.cmet.2024.10.015 39561764

[B129] Zong Z. Xie F. Wang S. Wu X. Zhang Z. Yang B. (2024). Alanyl-tRNA synthetase, AARS1, is a lactate sensor and lactyltransferase that lactylates p53 and contributes to tumorigenesis. Cell 187 (10), 2375–2392.e33. 10.1016/j.cell.2024.04.002 38653238

